# The outer-membrane export signal of *Porphyromonas gingivalis* type IX secretion system (T9SS) is a conserved C-terminal β-sandwich domain

**DOI:** 10.1038/srep23123

**Published:** 2016-03-23

**Authors:** Iñaki de Diego, Miroslaw Ksiazek, Danuta Mizgalska, Lahari Koneru, Przemyslaw Golik, Borys Szmigielski, Magdalena Nowak, Zuzanna Nowakowska, Barbara Potempa, John A. Houston, Jan J. Enghild, Ida B. Thøgersen, Jinlong Gao, Ann H. Kwan, Jill Trewhella, Grzegorz Dubin, F. Xavier Gomis-Rüth, Ky-Anh Nguyen, Jan Potempa

**Affiliations:** 1 Proteolysis Lab; Department of Structural Biology; “María de Maeztu” Unit of Excellence; Molecular Biology Institute of Barcelona, CSIC; Barcelona Science Park, Barcelona, Spain; 2 Department of Microbiology, Faculty of Biochemistry, Biophysics and Biotechnology, Jagiellonian University, Krakow, Poland; 3 Małopolska Centre of Biotechnology, Jagiellonian University, Kraków, Poland; 4 Department of Oral Immunology and Infectious Diseases, University of Louisville School of Dentistry, Louisville, KY, USA; 5 Department of Molecular Biology and Genetics and Interdisciplinary Nanoscience Center, Aarhus University, Aarhus, Denmark; 6 Faculty of Dentistry, University of Sydney, Sydney, Australia; 7 Institute of Dental Research, Westmead Centre for Oral Health and Westmead Institute for Medical Research, Sydney, Australia; 8 Faculty of Science, University of Sydney, Sydney, Australia

## Abstract

In the recently characterized Type IX Secretion System (T9SS), the conserved C-terminal domain (CTD) in secreted proteins functions as an outer membrane translocation signal for export of virulence factors to the cell surface in the Gram-negative *Bacteroidetes* phylum. In the periodontal pathogen *Porphyromonas gingivalis*, the CTD is cleaved off by PorU sortase in a sequence-independent manner, and anionic lipopolysaccharide (A-LPS) is attached to many translocated proteins, thus anchoring them to the bacterial surface. Here, we solved the atomic structure of the CTD of gingipain B (RgpB) from *P. gingivalis*, alone and together with a preceding immunoglobulin-superfamily domain (IgSF). The CTD was found to possess a typical Ig-like fold encompassing seven antiparallel β-strands organized in two β-sheets, packed into a β-sandwich structure that can spontaneously dimerise through C-terminal strand swapping. Small angle X-ray scattering (SAXS) revealed no fixed orientation of the CTD with respect to the IgSF. By introducing insertion or substitution of residues within the inter-domain linker in the native protein, we were able to show that despite the region being unstructured, it nevertheless is resistant to general proteolysis. These data suggest structural motifs located in the two adjacent Ig-like domains dictate the processing of CTDs by the T9SS secretion pathway.

Protein secretion onto the cell surface or into the extracellular milieu is essential for the survival of living organisms. In the case of diderm bacteria, secreted proteins must traverse two protein-impermeable lipid barriers: the inner- and outer-membranes (IM and OM, respectively), which are integral parts of the cell envelope in Gram-negative bacteria. To achieve this, diderm bacteria have evolved eight unique Types (I to VIII) of Secretion System (T*x*SS) to translocate cargo proteins to the cell surface, including surface appendages^1^. Cargo proteins either translocate across both cell envelopes directly from the cytoplasm (T1SS, T3SS, T4SS, and T6SS), or are first exported across the IM into the periplasm via signaling through an N-terminal signal peptide to the Sec translocon or twin-arginine translocation (Tat) systems and then transported through the outer membrane (T2SS, T5SS, T7SS, and T8SS). Subsequently, the cargo proteins become components of surface superstructures or surface proteins by remaining attached to the bacterial cell surface, or are released in a soluble form into the environment.

With the exception of T1SS and T2SS, which are widely distributed among prokaryotes, all other types of secretion systems are limited to proteobacteria with scattered occurrence in a few evolutionary lineages of bacteria^2^. This is the case for *Porphyromonas gingivalis*, a bacterium belonging to the *Bacteroidetes* phylum recognized as a major periodontal pathogen^3^. The virulence of *P. gingivalis* is dependent on secreted cysteine proteases called gingipains (RgpA, RgpB, and Kgp) that are essential for corruption of the host immune system and acquisition of nutrients and heme – a vital growth factor and source of black pigmentation of colonies on blood agar^4^. Gingipains are synthesized with a typical N-terminal signal peptide targeting them to the Sec translocon^5^,^6^ and are exported through the OM via a novel secretion system referred to as “Por Secretion System; PorSS” or following the naming convention, Type IX Secretion System (T9SS)^7^. Currently, the T9SS is composed of several OM, periplasmic, and IM proteins whereby knockout of any of these proteins leads to a gingipain-null, non-pigmented mutant phenotype in which inactive gingipains accumulate in the periplasm^7^,^8^,^9^,^10^,^11^,^12^.

Translocation of gingipains from the periplasm across the OM is dependent on a conserved C-terminal domain (CTD), which appears to be important for secretion of the proteins^13^ and in particular, truncation of the last few C-terminal residues of this domain leads to accumulation of gingipains in the periplasm^14^. Subsequently, the T9SS targeting signal was demonstrated to reside within the last 22 residues at the C-terminus of the CTD^15^. During gingipain translocation across the OM, the CTD is cleaved off by PorU, an essential component of the T9SS that acts as a sorting protease^16^, and anionic-LPS (A-LPS) is then covalently attached for surface anchorage to the OM^17^. In the case of RgpB, covalent attachment of A-LPS depends on the length of a linker sequence between the CTD and the preceding immunoglobulin-superfamily domain (IgSF), and occurs concurrently with the proteolytic cleavage of the CTD^18^.

Apart from gingipains, 27 other proteins in *P. gingivalis* possess CTDs, several of which were shown to be post-translationally modified during secretion via T9SS^19^. Together, they form an electron-dense layer of proteins highly enriched in gingipains on the *P. gingivalis* cell surface^20^. The *Tannerella forsythia* surface proteins TsfA and TsfB are secreted and processed in a similar manner (CTD cleavage and glucan attachment) via a T9SS, forming the semi-crystalline S-layer on the bacterial surface^21^,^22^. Several other CTD-bearing proteins, including KLIKK proteases^23^, are also secreted by *T. forsythia* via T9SS^24^,^25^. Collectively, S-layer and secreted CTD proteins regulate the virulence potential of *T. forsythia*, which together with *P. gingivalis* and *Treponema denticola*, are grouped into the so-called “red complex” organisms^26^. Red complex pathogens are instrumental in causing dysbiosis in the dental microbiota, which eventually leads to the development of chronic periodontitis in susceptible individuals^27^.

T9SS is not limited to periodontal pathogens and is widespread among members of the *Bacteroidetes* phylum with gliding motility^27^. In *Cytophaga hutchinsonii* and *Flavobacterium jonsoniae*, T9SS is required for secretion of cell surface gliding motility adhesins and cell surface-associated enzymes required for the digestion of crystalline cellulose and chitin^28^,^29^,^30^,^31^,^32^. As in periodontal pathogens, all proteins secreted by gliding *Bacteroidetes* via T9SS contain a CTD encompassing approximately 60 to 100 amino acid residues that is proteolytically cleaved during or after translocation across the OM^19^.

A fast growing body of evidence indicates that T9SS is used by diverse members of the *Bacteroidetes* phylum to secrete CTD-bearing proteins. Despite limited sequence identity, CTDs share some sequential motifs that maybe important in its function. However, not much else is known about the structure of this domain. To elucidate the role of the CTD in RgpB secretion and posttranslational modification, we solved the crystal structure of the CTD alone and in tandem with the preceding IgSF domain. This, and the known structure of mature RgpB in complex with the N-terminal prodomain, allowed us to reconstruct the structure of the RgpB zymogen: the catalytic domain followed by tandem of Ig-like folded domains. Furthermore, small angle X-ray scattering (SAXS) revealed no fixed reciprocal orientation of the IgSF and CTD domains, which are connected by an unstructured linker. Finally, the propensity of the CTD to dimerize may represent a mechanism involved in domain recognition by T9SS machinery.

## Results

### Structure of the rIgSF-CTD tandem

Whereas the structures of mature RgpB alone and in the inhibitory complex with the N-terminal pro-domain are available^33^,^34^, there is no structural data available on the CTD of proRgpB or any other T9SS-translocated protein. This domain is conserved in all proteins secreted by T9SS to signal their translocation across the outer membrane. To cast light on the structure-function relationship of the CTD, we recombinantly expressed this domain alone or together with the preceding IgSF domain (rIgSF-CTD) as GST fusion proteins carrying a Prescission cleavage site. In addition, our previous report described the strange behavior of non-glycosylated RgpB production in the native organism when the junction between the IgSF and CTD was lengthened by insertion of a hexapeptide motif^18^, to investigate this phenomenon, we also produced recombinant IgSF-CTD with insertion or substitution of hexapeptide motifs in the linker region for analysis.

Despite extensive efforts, we were unable to crystallize the wild-type version of recombinant protein containing the wild-type IgSF-CTD (residues T^577^–K^736^; for residue numbering, see UniProt database entry P95493). However, diffraction quality crystals were obtained using mutants of the same construct, which included an inserted hexahistidine after residues S^664^ or I^665^ (recombinant products r664i6H and r665i6H, respectively), or by substitution of six consecutive linker residues (I^665^ADVAN^670^) with a factor Xa cleavage motif (IEGRAA; r665sXa). The three structures were solved and refined with diffraction data to 1.9 Å, 2.4 Å, and 2.1 Å resolution, respectively (Table S1). Each variant of rIgSF-CTD formed nearly identical crystal structures (RMSD of 0.760 Å for r665i6H and 0.327 Å for r665sXa, with respect to r664i6H for 298 aligned residues). Thus, we performed our analysis with the structure refined to highest resolution (r664i6H).

**Figure 1:**
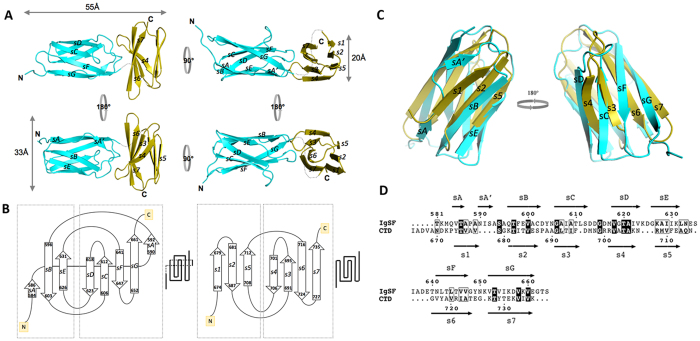
Structure and topology diagram of the rIgSF-CTD tandem and structural superposition of the IgSF and CTD domains calculated using the Secondary Structure Matching (SSM) algorithm^66^. (**A**) Overview of the IgSF (cyan)-CTD (yellow) tandem. The structure is elongated, with both domains packing in a perpendicular fashion. The shape of the IgSF closely resembles a β-barrel, whereas the CTD is more a regular β-sandwich. (**B**) Topology and schematic diagrams of, respectively, IgSF (left) and CTD (right). Although both domains have an Ig-like fold, significant differences are observed between sheet composition. Whereas the N-terminal sheet of each domain is antiparallel in both cases, the C-terminal sheet differs, being mixed in IgSF. In IgSF, strands sA-sA’ and, to a lower extent, sD, are establish interactions with both sheets of the barrel, thus generating a higher curvature in the surface of the sheets. (**C**) IgSF (cyan, sA–sG) and CTD (yellow, s1–s7) can be superimposed with an RMSD of 2.1 Å for 63 aligned residues, thus confirming that both fragments have an Ig-like fold. However, main differences occur at the first strand (sA–sA’ for IgSF, s1 for the CTD) and in the curvature of the sheets (less prominent for the CTD). (**D**) Structure-based sequence alignment. Despite low sequence identity, the number, length and type of secondary structures reveals that both are Ig-like domains.

The recombinant r664i6H product (Table S1) assembled into a monoclinic lattice, producing crystals belonging to monoclinic space group P2_1_. The asymmetric unit contained two molecules, each corresponding to a r664i6H tandem with approximate dimensions of 55 Å × 33 Å × 20 Å (Fig. 1A). In each tandem, the IgSF and CTD domains showed an orthogonal arrangement, with their longest axes tilted by 90 degrees with respect to each other. Regions with defined electron density included the residues V^579^-G^662^ (IgSF) and K^672^–K^736^ (CTD). The 9-residue linker (T^663^-D^671^) between the immunoglobulin-like subdomain and the CTD did not yield visible electron density, indicating that this fragment was disordered in the crystals. The hexahistidine tag was inserted in this disordered area.

The IgSF domain consists of a seven-stranded β-barrel composed of two β-sheets (sA-sG, with the first strand split into two). The antiparallel smaller N-terminal sheet is initiated with a short strand, sA (Q^584^-T^586^), which transitions to a connecting loop leading to strand sA’, which becomes a peripheral, parallel strand in the larger C-terminal 5-stranded β-sheet (Fig. 1A,B). Strand sA’ then folds back to connect with sB, the central strand of the N-terminal sheet. sB progresses via a loop to sC, which forms a β-hairpin and then continues to sD within the C-terminal sheet. This creates the inner core of the Greek-key motif. Turns after sD and sE, with the latter forming a long loop to reach sF, complete the three-stranded, N-terminal sheet. The sF strand is connected by a β-hairpin motif with sG, which completes the IgSF domain. No disulfide bridges are observed within this domain, which is assembled into a cylindrical β-barrel due to the interactions between the lateral strands of both sheets. This is clear at the sA-sA’ strand, which is split and interacts with the two β-sheets, and also at the sD strand, although to a lower extent. Together, these interactions generate a clamping force able to increase the curvature of the outer surface of the barrel.

The overall fold of the rCTD distantly resembles that of the IgSF domain, and both domains superimpose with an RMSD of 2.11 Å for 63 aligned residues (Fig. 1C), despite sharing a sequence identity below 20% (Fig. 1D). As the IgSF domain, the CTD is composed of two almost completely parallel to each other β-sheets that are formed by three and four antiparallel strands (Fig. 1A). This differs with respect to the IgSF domain, in which the overall shape somewhat resembles a flattened ellipsoid. In the CTD, all β-strands are of similar length and no significant interactions occur between the lateral strands of each sheet, thus generating a compact, sandwich-like structure instead of a β-barrel. In comparison to the IgSF domain, the β-turns/hairpins and connecting loops are shorter in the CTD, and the sheets are more regular and clearly identifiable. Nevertheless, the structure also follows a Greek-key topology typical of Ig-like folded domains (Fig. 1A,B, right panel).

The CTD has maximal dimensions of 20 Å × 32 Å × 15 Å. It starts with strand s1, which, after a β-hairpin, continues to strand s2, followed by a loop that crosses to the other side of the sandwich, becoming s3 of the C-terminal sheet (Fig. 1B, right panel). The s3 strand makes a β-hairpin-like loop and progresses to strand s4, thus constituting the inner motif of the Greek-key, similar to the IgSF domain. The s3–s4 loop contains D^696^, in which the side chain forms double hydrogen bonds with the α-nitrogens of G^699^ and R^700^ of the polypeptide backbone (that in this area runs orthogonally to s3), generating a curved hairpin resembling a spoon (Fig. 1A). At the tip of this spoon structure, a methionine protrudes from the surface. A similar structure occurs in the loop that connects s4 of the C-terminal sheet with s5, the last strand of the N-terminal sheet. Within this loop, double hydrogen bounds between N^707^ and the α-nitrogens of S^687^ and A^690^ produce a structure that resembles a highly curved antiparallel, double-stranded sheet. The long loop joins s5 and s6 within the C-terminal sheet, then the polypeptide chain connects via a β-turn with s7, the last and longest strand of the domain, leading to the end of the protein. The two hairpin motifs (strands s1–s2 and s6–s7) are exposed in opposed sides along the longest axis of the CTD.

It can be speculated that the unusual conformation of the s3–s4 and s4–s5 loops, along with conservation of D^696^ followed by methionine or branched aliphatic amino acids (valine, leucine and isoleucine) in T9SS cargo proteins (Fig. S1) might be involved in the function of the CTD in domain sorting proteins for secretion via T9SS. Nevertheless, mutation of D^696^ in RgpB does not affect gingipain secretion or posttranslational modification^18^.

Structural similarity searches using the CTD domain identified the human surface antigens CD48 (PDB 2DRU; DALI Z-score = 8.5; RMSD = 2.1 Å; length of alignment = 64; sequence identity = 13%), CD58 (1CCZ; 8.4; 2.1; 64; 13), CD2 (1HNG; 8.3; 2.1; 64; 13), and CD4 (1CID; 8.2; 2.7; 63; 16), all of which possess an Ig-like fold. This result confirms again that the CTD belongs to the Ig-like superfamily, making the IgSF-CTD arrangement a tandem of Ig-like domains.

### Small-angle X-ray scattering (SAXS) structure of rIgSF-CTD tandem

**Figure 2:**
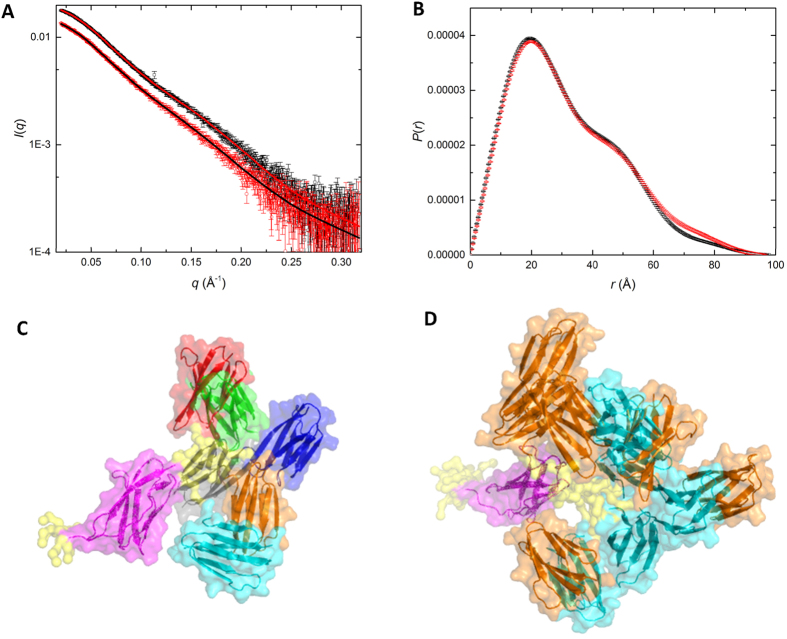
Small angle X-ray scattering (SAXS) of rIgSF-CTD. **(A**) SAXS data for wild-type rIgSF-CTD (black symbols) and the r664i6H variant (red symbols) with the ensemble optimisation model fit shown as lines in the reverse colours. (**B**) Atom pair distribution, *P*(*r*) versus *r*, calculated as the Fourier transform of the *I*(*q*) versus *q* profiles in A, with the same colour coding. (**C**) Ensemble optimisation modelling (EOM) selected representative structures aligned via the IgSF domains (magenta) with the small flexible tip of N-terminal residues and the linker in yellow (surface representation only), showing the range of positions for the CTD domains within the wild-type rIgSF-CTD construct; the CTD domains coloured differently for each conformation within the set. (**D**) Overlay of EOM selected sets for wild-type rIgSF-CTD (cyan CTD domains) and r664i6H variant (orange CTD domains) to show the range of flexibility of the CTD with respect to the aligned IgSF domain (magenta).

Small-angle X-ray scattering (SAXS) data from recombinant rIgSF-CTD and the r664i6H variant (Fig. 2) indicate that in solution the two domains are connected by a flexible linker. The small-angle scattering for both constructs indicate they are pure monomers in solution (see Methods) and thus the SAXS data are suitable for structural analysis (Table S2). Inverse Fourier transform of *I*(*q*) versus *q* to yield the density-weighted atom-pair length distribution (*P*(*r*) versus *r* (Fig. 2B) shows an asymmetric profile with a peak at ∼20 Å, a shoulder around 50 Å and a maximum dimension (*d*_*max*_) ∼100 Å, consistent with an elongated, two-domain structure. As might be expected, the *Rg* value for the wild type construct (25.2 ± 0.1 Å) is somewhat smaller than that for the r664i6H variant (26.0 ± 0.2 Å) containing the hexahistidine insertion, but there appears little difference in *d*_*max*_ values (97–98 Å) and the overall *P*(*r*) profile is very similar, with some small variation above *r* values of 60 Å.

Modelling against the scattering data for each of the two constructs using dummy residues to define a volume occupied by each structure shows an extended asymmetric shape (Fig. S2) consistent with the expected two domains and the *P*(*r*) analysis. Atomistic models were created to test against the scattering data using the two structured domains from the crystal structure (IgSF residues 579–662 and CTD residues 672–736) and a linker of undefined structure for the wild type (residues 663–671) and r664i6H variant (residues 663–671 plus the hexahistidine insertion). An optimised average best-fit structure was generated (using the program BUNCH) that yielded a χ^2^ value for the model fits to the scattering data of 1.3 (Table S2) and models that showed few or no interactions between the domains (Fig. S2). Multiple runs of BUNCH produced similar fits, with varying orientations of the IgSF and CTD domains for both structures.

SAXS data yield structural information on the time and ensemble average conformation in solution, and to test the possibility that the domain linker region might be flexible in solution, an ensemble optimisation modelling (EOM) approach was used with the same two domain structures and flexible linkers. The EOM calculations for both constructs gave a significantly improved fit to the data with a χ^2^ values of 1 (Table S2) and an excellent fit to the data over the measured *q*-range (Fig. 2A). Examination of the *R*_*g*_ and *d*_*max*_ population distributions for the two sets of ensembles (Fig. S3) shows a weighting toward more compact structures with *R*_*g*_ values ∼2–3 Å smaller than the average values, but a significant population of more extended conformations are also observed. Further, the hexahistidine insertion in the r664i6H variant provides for greater range of *R*_*g*_ and *d*_*max*_ values in the ensemble; by ∼4 Å in *R*_*g*_ and >10 Å for *d*_*max*_. Figure 2C shows the selected representative example structures used to define the ensemble in the wild-type rIgSF-CTD construct. The scattering data are consistent with the hexahistidine insertion in the r664i6H construct having extended the overall length of native linker between the IgSF and CTD domains and thus facilitating a wider sampling of conformational space in the EOM results (Fig. 2D), perhaps enabling the inter-domain contacts that aided in crystallisation of this variant.

### Structure of a domain-swapped rCTD dimer

**Figure 3:**
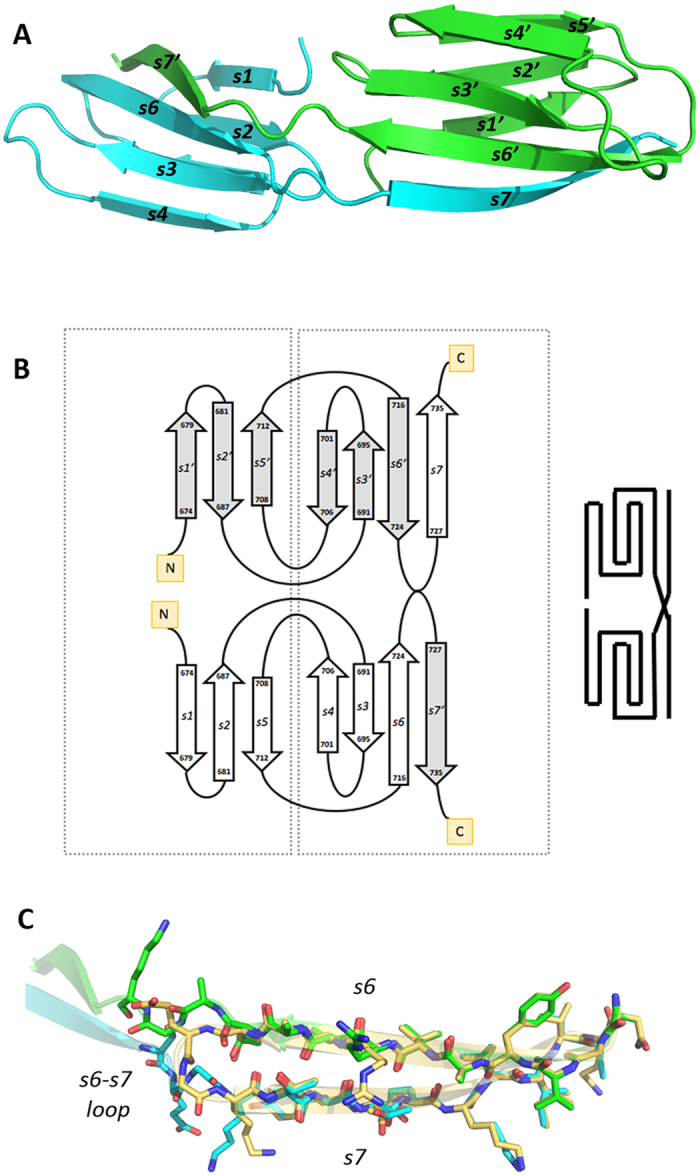
The rCTD structure. (**A**) Purified rCTD yielded a dimeric structure (chains in cyan and green), in which both domains interact head to back. The C-terminal strand does not fold back, running antiparalel to the penultimate strand but rather runs in extended conformation and provides the last strand of the symmetric partner. (**B**) Topology diagram showing the domain swap of the C-terminal strands. (**C**) Stick model showing the superposition of strands s6 and s7 in the CTD of the IgSF-CTD tandem structure (carbon atoms in gold) and in the dimeric standalone CTD structure (strand s6 of one monomer in green, strand s7 of the other monomer in cyan). Except for the respective linkers to the preceding strands s6, the two s7 strands nicely fit on top of each other.

In addition to the rIgSF-CTD tandem of the Ig-like folded domains, we solved the structure of the rCTD alone. Crystals were obtained from a fragment encompassing residues S^664^–K^736^, which crystallized in the hexagonal space group, P6_5_ (Table S1). The structure was solved to 1.9 Å resolution (Table S1) and the Fourier map enabled us to trace residues K^672^–K^736^. The asymmetric unit contained a dimer of rCTD molecules (approximately 75 Å × 21 Å × 14 Å) in a side-to-side arrangement, with the monomers related to each other by a twofold axis. The interface between the monomers had a total surface area of 1,440 Å, accounting for 28% of the total accessible surface area of the dimer. Superimposition of one monomer with the corresponding domain in the rIgSF-CTD tandem revealed that the conformations of strands s1–s6 (corresponding to the 55 N-terminal residues) of the rCTD were very similar (Fig. 3). In both structures, these strands constitute a parallel sandwich of two three-stranded β-sheets with the fully conserved inner Greek-key topology, including the same β-turns/hairpins and connecting loops (Fig. 3B). However, a significant difference was observed between the polypeptide chain encompassing residues V^717^–V^735^ corresponding to the s6-s7 loop and the last strand (s7) of the CTD. In this structure, strand 7 was swapped between the two monomers and constituted the primary interface between them. Thus, in this conformation, the s6-s7 loop, instead of a turn, adopts an extended conformation along the s6 main axis, bringing s7 to interact with the neighboring monomer. This change rotates the position of E^725^ side chain by almost 180 degrees, and repositions T^724^, which is able to establish both main and side chain H-bonding interactions with the backbone of K^727^ from the neighboring monomer, thus stabilizing this new conformation (Fig. 3C). The equivalent region from the neighboring monomer then occupies the space generated by this movement in an identical manner. This domain swap, as a consequence, generates an antiparallel β-sheet between strands s6 and s7, as occurs in the absence of swapping, that has the same backbone and side chain set of interactions (see Fig. 1A), with the only differences being the monomer that provides s7 and the conformation of the s6-s7 loop. Minor variations occur on the surface side chains of T^730^ and R^721^ due to the difference in content and packing of the crystals.

### Probing rIgSF-CTD and its variants by limited proteolysis

**Figure 4:**
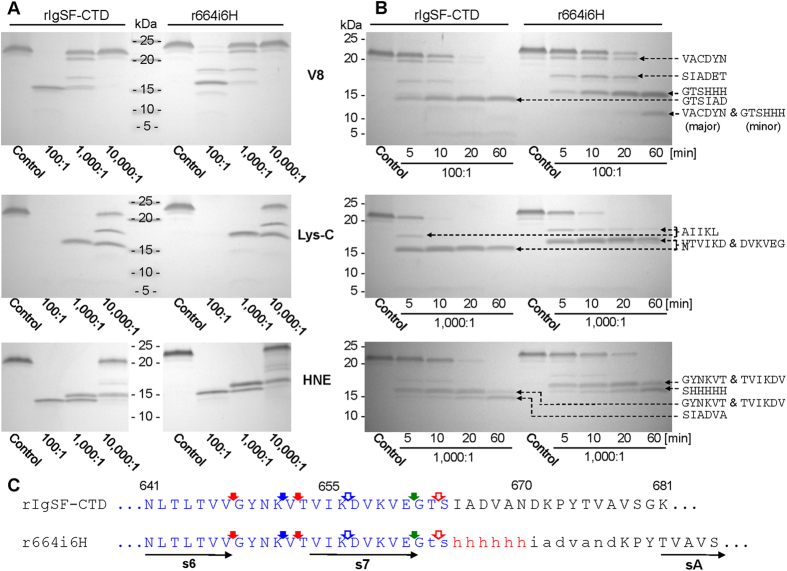
Probing the structure of the wild-type rIgSF-CTD and the r665i6H variant by limited proteolysis. Both proteins were incubated with V8 protease (V8), Lys-C endopeptidase (Lys-C) and human neutrophil elastase (HNE) at (**A**) different substrate:enzyme molar ratios (concentration-dependent proteolysis) for 1 h or (**B**) at a constant 100:1 substrate:enzyme ratio for different time intervals (time-dependent proteolysis). Boiling with SDS-PAGE reducing sample buffer terminated reaction and samples were subjected to SDS-PAGE, transferred onto PVDF membrane for N-terminal sequencing (sequences next to each panel). (**C**) Identified primary cleavage sites within the linker region are indicated by solid arrows: red for elastase, blue for Lys-C and green for V8 protease. Open arrows indicate secondary cleavages by the respective enzymes. Polypeptide sequence comprising the IgSF domain is in blue font; the linker region between IgSF and CTD domains is in lowercase and inserted 6×His is in red. Horizontal arrows below the sequence indicate β-strands in the IgSF-CTD tertiary structure.

Limited proteolysis is a technique commonly used to determine domain composition and probe for structural differences induced by physiological and/or pathological modifications^36^. We applied this method to elucidate differences in the conformation of the linker between the IgSF and CTD domains, which may explain the failure of the native rIgSF-CTD form to crystallize. To this end, we probed r664i6H and native rIgSF-CTD by limited proteolysis using V8 protease, Lys-C endopeptidase, and human neutrophil elastase (HNE) (Fig. 4A,B). In both cases, the same peptide bonds within the IgSF-like domains were cleaved, but with different kinetics. Apart from E^599^#V, E^634^#S, and K^627^#A cleavages (“#” indicates hydrolyzed peptide bonds) away from the IgSF-CTD junction, all other major primary cleavages were mapped to a loop between strands s6 and s7 and directly at the end of s7 (Fig. 4C). Proteolysis of Lys-Asp within strand s7 and cleavage of Thr-Ser peptide bonds by Lys-C peptidase and HNE, respectively, appear to be secondary to upstream cleavages at Lys-Val, Val-Gly, and Val-Thr. Interestingly, no proteolysis occurred directly within the flexible linker loop, despite the presence of several peptide bonds (Ile-Ala, Ala-Asp, Val-Ala, and Ala-Asn) that should be susceptible to proteolysis by HNE.

**Figure 5:**
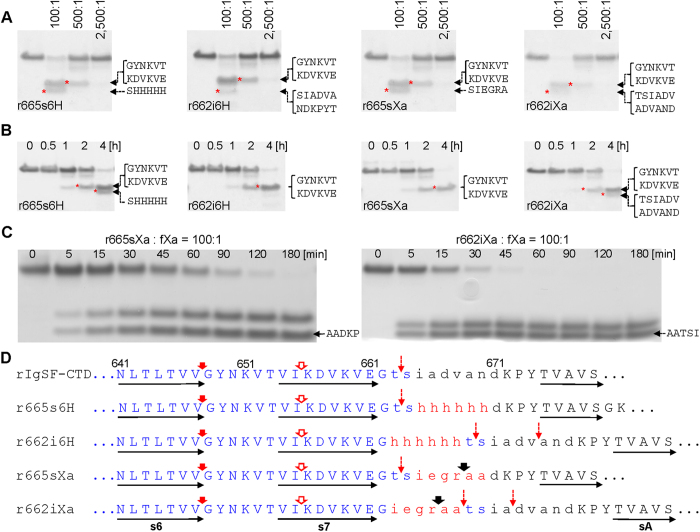
Probing the structure of insertion/substitution variants of IgSF-CTD by limited proteolysis. IgSF-CTD variants were incubated with (panels **A** and **B**) porcine pancreatic elastase (PPE) or (panel **C**) factor Xa (fXa) at different substrate:enzyme molar ratios (concentration-dependent proteolysis) for 1 h or at a constant 100:1 substrate:enzyme ratio for different time intervals (time-dependent proteolysis). Boiling with SDS-PAGE reducing sample buffer terminated reaction and samples were subjected to SDS-PAGE, transferred onto PVDF membrane for N-terminal sequencing (sequences next to each panel). (**D**) Identified primary cleavage sites within the linker region are indicated by solid arrows: red for PPE and black for fXa. Open and broken red arrows indicate secondary and minor cleavage sites for PPE. Polypeptide sequence comprising the IgSF domain is in blue font; the linker region between IgSF and CTD domains is in lowercase with inserted/substituted sequences in red font. Horizontal arrows below the sequence indicate β-strands in the IgSF-CTD tertiary structure.

Since the first round of probing by limited proteolysis did not reveal any cleavage events within the inter-domain linker, we constructed and analyzed three additional variants of rIgSF-CTD for susceptibility to cleavage by porcine pancreatic elastase (PPE) and factor Xa: substitution of residues I^665^ADVAN^670^ with hexahistidine residues in r665s6H; and insertion of hexahistidine or Xa cleavage sequence (IEGRAA) after residue Gly^662^ in recombinant proteins r662i6H and r662iXa, respectively (Fig. 5D). Interestingly, the protein variants with insertions elongating the inter-domain linker were cleaved faster by PPE than those for which substitution altered the sequence but not the length of the linker region (Fig. 5A,D). Primary cleavage by PPE occurred at the end of strand s6 (Val-Gly) followed by hydrolysis of the Ile-Lys peptide bond. All cleavages downstream of strand s7 were secondary (see Fig. 5A, dashed arrows) and most likely represent truncation of CTD. Again, despite the presence of many peptide bonds potentially susceptible to proteolysis by PPE (Ile-Ala, Ala-Ala, Ala-Asp, Val-Ala, Ala-Asn) in the junction region of the native IgSF-CTD protein, none were initially cleaved by PPE. In addition, the same bonds shifted downstream by insertion of 6×His (r662i6H) or the factor Xa cleavage motif (IEGRAA; in construct r662iXa) were also resistant to proteolysis by PPE. Furthermore, potential PPE cleavage sites (Ala-Ala, Ala-Asp, and Ala-Thr) within the IEGRAA sequence in r662sXa and r664iXa were secondary PPE cleavage sites in proteins with the insertion of the sequence, but not in the protein in which it was introduced by substitution. The lack of cleavage observed within the linker by elastase is clearly not due to inaccessibility of this loop to proteolytic enzymes, as proteins bearing the IEGRAA motif by insertion/substitution were efficiently cleaved at the Arg-Ala peptide bond by fXa (Fig. 5B,D). Interestingly, however, there was a clear difference in the kinetics of cleavage events, with r662iXa being far more susceptible to proteolysis than r665sXa (Fig. 5C).

Taken together, these results demonstrate that insertion and substitution within the connecting linker do not affect domain structure or inter-domain arrangement, and suggest that the linker is resistant to proteolysis despite the presence of peptide bonds potentially susceptible to cleavage by HNE and PPE. Instead, these two proteases very efficiently cleave the native and mutated rIgSF-CTD upstream within a loop between s6 and s7. Conversely, the intra-domain loop in mutants bearing the IEGRAA motif was highly susceptible to proteolysis by fXa, a protease with unique specificity^37^.

### Probing full-length proRgp for proteolytic release of CTD

In stark contrast to the wild-type W83 strain which retains anionic-LPS conjugated RgpB on the OM, *P. gingivalis* mutants expressing RgpB with insertion of a hexahistidine-tag or an 8-residue StrepII^©^ tag after residue G^662^ in the junction of IgSF-CTD secrete the RgpB into the culture medium in a soluble, non-glycosylated form^18^. Conversely, replacement of residues S^664^–N^670^ with Ala-6×His yielded a mutant with wild-type phenotype. The effect of these and other mutations in the linker region between the IgSF and CTD domains on RgpB secretory phenotype suggests that the length of the junction is critical for RgpB glycosylation^18^. This observation was confirmed presently using IEGRAA as the motif for insertion after residue G^662^ of RgpB in *P. gingivalis* (mutant RgpB662iXa6H) resulting in secretion of soluble, non-glycosylated RgpB or substitution of residues I^665^–N^670^ (RgpB665sXa6H) with IEGRAA resulting in glycosylated, membrane-bound RgpB (Fig. S4).

**Figure 6:**
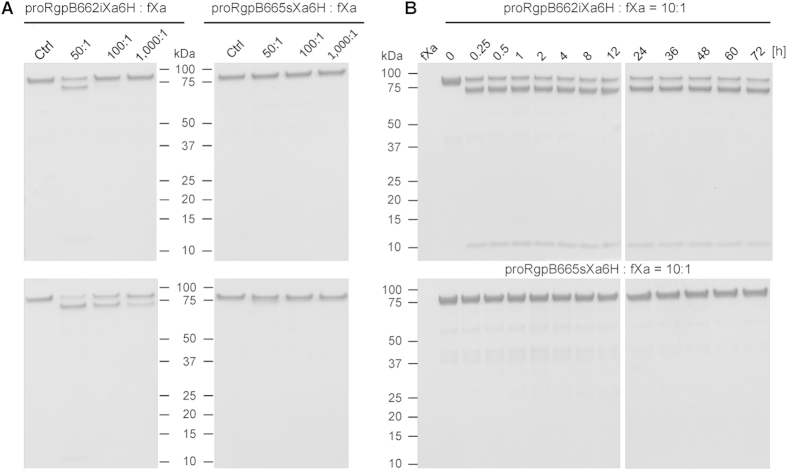
Probing of full-length proRgpB variants expressed in the native organism bearing factor Xa cleavage motif (IEGRAA) in the junction between the IgSF and CTD domains. (**A**) Concentration-dependent and (**B**) time-dependent limited proteolysis with factor Xa. (A) 1.67 μM of proRgpB with insertion (RgpB662iXa6H) or substitution (RgpB665sXa6H) of residues in the linker region with Xa cleavage motif were incubated either alone or with fXa at the molar ratio 50:1, 100:1 and 1,000:1 at 37 °C for 3 h (upper panels) or 15 h (lower panels). The proteolysis was assessed by SDS-PAGE. Ctrl, control of proRgpB variant incubated alone. (**B**) ProRgpB variants were incubated at 37 °C with fXa at 10:1 ratio for up to 72 h. At indicated time intervals, samples were withdrawn and analyzed by SDS-PAGE for proteolytic degradation.

Based on subtle but clear differences in the kinetics of cleavage of IgSF-CTD carrying an insertion vs. a substitution, we hypothesize that the rate of proteolysis of the CTD during the secretion process is responsible for the lack of glycosylation of insertion-carrying RgpB. To further investigate this possibility, we purified full-length, C-terminally hexahistidine-tagged proRgpB in which the IEGRAA motif was either inserted in the IgSF-CTD linker (RgpB662iXa6H) or replaced residues I^665^–N^670^ with the motif (RgpB665sXa6H). The mutated RgpB variants were expressed in the Δ*porN* secretion-deficient mutant of *P. gingivalis* W83 to allow accumulation of full-length proRgpB in the periplasm and for purification by affinity chromatography on Ni^2+^-Sepharose. The proRgp variants were then subjected to concentration- (Fig. 6A) and time-dependent (Fig. 6B) digestion with fXa. Interestingly, while the proRgpB from RgpB662iXa6H was susceptible to proteolysis by fXa, proRgpB from RgpB665sXa6H was completely resistant even after 72 h incubation at a ratio of 10:1 with fXa (Fig. 6B, lower panel). Further, although most of proRgpB from RgpB662iXa6H was cleaved very rapidly (at a substrate:fXa ratio of 10:1), the remaining protein resisted proteolysis, regardless of incubation time. This suggests that proRgpB from RgpB662iXa6H occurs in two different forms: one resistant to proteolysis by fXa and the other susceptible. Cumulatively, our findings suggest that the rate of proteolytic cleavage of the IgSF-CTD linker region may influence the process of glucan attachment to the translocating RgpB with rapid cleavage resulting in the non-glycosylated form of the protein.

### Confirmation of CTD dimerization phenomenon

**Figure 7:**
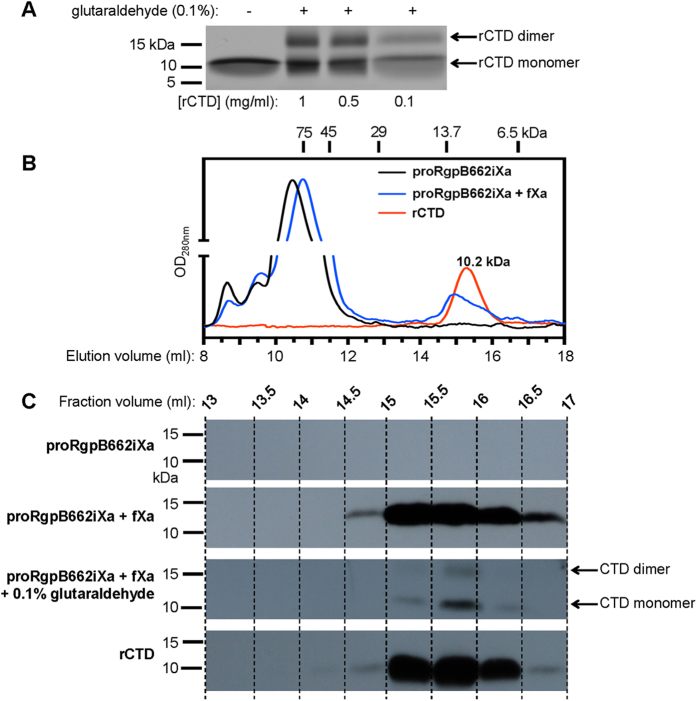
Soluble rCTD is a dimer in equilibrium and the CTD cleaved off natively expressed proRgpB spontaneously dimerizes. (**A**) rCTD at 1, 0.5 and 0.1 mg ml^−1^ was treated with glutaraldehyde and analysed by SDS-PAGE. (**B**) Recombinant CTD (rCTD) (red), proRgpB662iXa (black), and proRgpB662iXa preincubated with fXa (blue) were subjected to size exclusion chromatography on a Superdex 75 10/300 GL column equilibrated with 50 mM Tris, 150 mM NaCl, 2.5 mM CaCl_2_, 0.02% NaN_3_ pH 7.5 (**C**) Indicated fractions of resolved proteins were analysed by Western blot using anti-rCTD antibodies to reveal the CTD content in each analysed fraction.

To confirm that the rCTD subjected to crystallization was indeed a stable dimer apparently formed by strand swapping, we exposed freshly extracted, serially diluted rCTD to chemical crosslinker, glutaraldehyde, followed by SDS-PAGE analysis for the presence of the dimer. This method is commonly used to assess protein oligomeric status^38^,^39^. As shown in Fig. 7A, regardless of the dilution, even at as low protein concentration as 0.1 mg ml^−1^, the clear presence of dimer was observed after cross-linking. To exclude the possibility that the observed dimer of rCTD after glutaraldehyde treatment was an artifact, rCTD and r664i6H at the same concentration (0.1 mg ml^−1^) were exposed to different glutaraldehyde concentrations (0.1, 0.05, 0.01%). Dimer formation was observed only in the rCTD sample (data not shown). Keeping in mind that cross-linking is never 100% efficient and the fact that only one form of rCTD was detected through gel filtration as represented by a single, symmetrical peak at 10.2 kDa (Fig. 7B), we interpret these data to be consistent with the rCTD existing predominantly as a dimeric form (theoretical molecular mass of 15.8 kDa) in solution.

Considering the structural similarities in the swapped area in the homodimer, formation of heterodimers with the IgSF domain, or even with another Ig-like domain, is conceivable, as this occurs in several other proteins with Ig-like folds^35^. Therefore, to explore this energetically affordable domain swapping as possible part of the mechanism of recognition and/or translocation of T9SS cargo proteins, we investigated propensity of CTD released from proRgpB produced in the native organism to dimerize upon cleavage. To this end, full-length, purified proRgpB bearing the factor Xa cleavable motif inserted in the linker between IgSF and CTD (proRgpB662iXa) was incubated with Xa, the digest was resolved on a gel filtration column and selected fractions were analyzed for the presence of the CTD. As shown in Fig. 7, both the elution profile (Fig. 7B) and the WB signal intensity profile (Fig. 7C) of the CTD released from proRgpB662iXa overlap very nicely with the profiles of the rCTD dimer. Moreover, when the CTD released from proRgpB662iXa was crosslinked with glutaraldehyde, the dimer band could also be detected (Fig. 7C). These observations that the CTDs can spontaneously swap their C-terminal β-strand may represent an important feature of the CTD which contributes to the recognition of cargo protein secretion by T9SS.

## Discussion

IgSF protein domains are widely distributed in living organisms with a general function related to protein-protein interactions, such as protein binding or molecular recognition processes^40^. Here, we show for the first time that the OM translocation signal, a conserved CTD of RgpB, has a typical Ig-like fold encompassing seven antiparallel β-strands organized in two β-sheets that are packed against each other in a β-sandwich (Fig. 1). Significantly, structure-based alignment of 32 CTDs identified or predicted in *P. gingivalis* (Fig. S1) shows that the (predicted) β-strands overlap perfectly with the conservative motifs identified through the structure of RgpB CTD: motif A is composed of strands 1 and 2, motif B contains strands 3 and 4, and motifs C,D, and E encompass strands s5, s6, and s7, respectively^13^,^14^. In β-strands, hydrophobic and hydrophilic residues occur in an alternating pattern, with hydrophobic side chains pointing towards the interior of the molecule. This pattern, especially in strands s2, s3, s5, and s6, is strongly conserved (Fig. S1). The structurally equivalent buried hydrophobic residues of these strands form a ring of hydrophobic interactions in the core of the domain and are engaged in folding of IgSF domains^41^. In addition, similar conserved motifs are also present in CTDs derived from other proteins secreted via T9SS by disparate Gram-negative bacteria: *T. forsythia, Prevotella intermedia*, and *Parabacteroides distasonis*^19^. This strongly implies that in addition to *P. gingivalis*, the CTD domains of other *Bacteroidetes* T9SS cargo proteins also share the common Ig-like fold despite having a negligible degree of identity with respect to the primary structure.

The C-terminus of the CTD has been reported to contain the export signal that targets the cargo protein for OM export through the T9SS system^14^. Indeed, fusing the last 22 residues of CTD from HPB35 – which overlap perfectly with the last two β-strands of the CTD – to a green fluorescent protein (GFP), resulted in its secretion and glycosylation in *P. gingivalis*^15^. Of note, this C-terminal export signal is the same region of the protein that strand swapping occurs in our rCTD dimer. Collectively, the data suggest that the hairpin motifs formed by the two highly conserved β-strands in the CTD C-terminus comprise the primary T9SS recognition signal.

**Figure 8:**
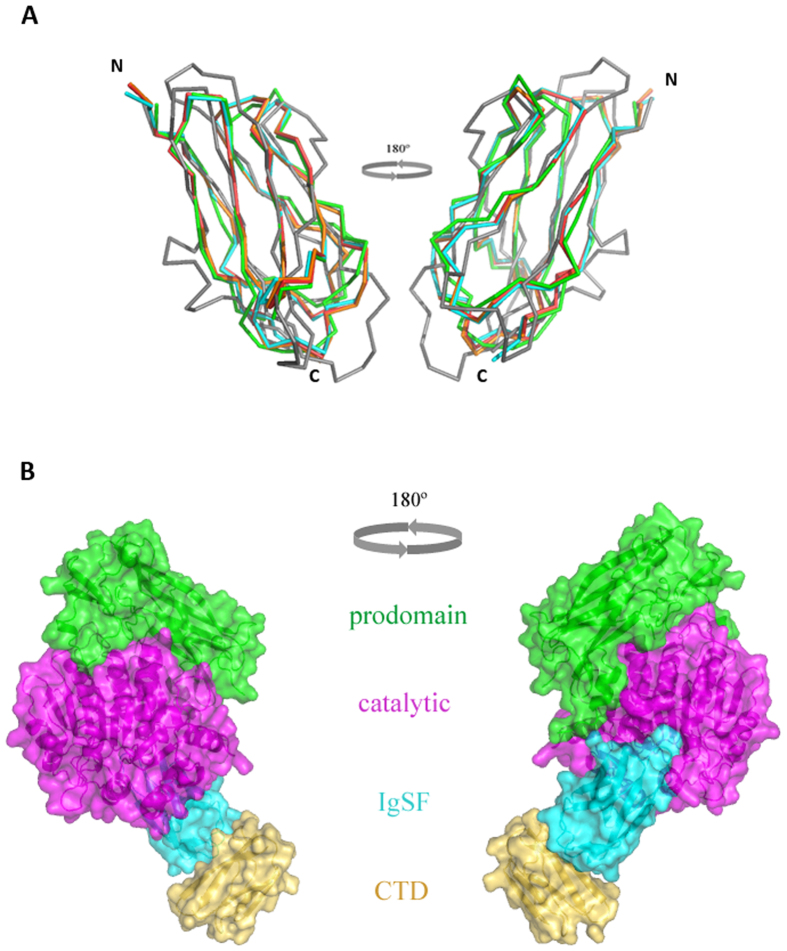
Superimposition of the IgSF domains in the presence of other domains in RgpB, and a model of the full-length latent proRgpB. (**A**) The IgSF domain of r664i6H (cyan) superimposes perfectly with the IgSF domain of mature RgpB (PDB id. 1CVR, in red) and RgpB-N-terminal prodomain complex (PDB id. 4IEF, in orange) and shows significant structural conservation with related IgSF sequences from Kgp (PDB id. 4RBM, in green) and PPAD (PDB id. 4YT9, in grey). (**B**) Surface representation of the chimeric, multi-domain model of the latent full-length proRgpB, transiently present in the periplasm during secretion, including the N-terminal prodomain (green), the catalytic domain (purple), the IgSF domain (cyan) and the CTD (yellow).

In RgpB, the IgSF domain directly follows the catalytic caspase-like catalytic domain. The IgSF structure in the mature RgpB^33^, in complex with the N-terminal prodomain of RgpB^34^, and in the present report adopts a nearly identical conformation. Taking as a reference the IgSF domain (579–661) from the latter structure, RMSDs of 0.456 Å (mature RgpB, 83 aligned residues) and 0.453 Å (proRgpB, 82 aligned residues) was calculated, demonstrating that neither the presence of the preceding catalytic domain nor the succeeding CTD induces any conformational change in the IgSF domain. This is clearly visible by the almost perfect superimposition of IgSF in RgpB and in IgSF-CTD structures (Fig. 8A). Therefore, by superpositioning of the N-terminal prodomain:RgpB complex onto the IgSF-CTD tandem, we constructed a model corresponding to the periplasmic form of RgpB, including the N-terminal prodomain, the catalytic domain, the IgSF and CTD domains, prior to entering the T9SS secretory pathway (Fig. 8B). This structural model suggests that the IgSF domain may act as a spacer or insulator between the catalytic and CTD domains. To date, the IgSF domains in T9SS secreted proteins have been structurally characterized in Kgp^42^, RgpB^33^,^34^, and PPAD^43^ are found to be similar to one another.

In the crystal structure of the rIgSF-CTD tandem of Ig-like folded domains, the interdomain linker was disordered, and was therefore expected to be highly susceptible to proteolysis. Contrary to this expectation, in both the native protein and recombinant proteins modified by insertions or substitutions, the wild-type linker was resistant to proteolysis (Figs 4 and 5). All primary cleavages occurred within the loop connecting β-strands 6 and 7 regardless of the linker modification. From these results, it can be inferred that the linker sequence, although lacking secondary structure by crystallography or SAXS of the recombinant rIgSF-CTD, the junction is impervious to proteolytic attack, suggestive of some kind of structural barrier. SAXS on proRgpB may be more informative in this regard and is currently being explored in our laboratories. The exception was cleavage by factor Xa of the linker engineered to contain an inserted hexapeptide motif (IEGRAA), which is recognized by this very specific protease. In this case, the protein containing the IEGRAA *insertion* was processed more rapidly than rIgSF-CTD carrying an IEGRAA *substitution*. Cleavage by fXa likely results from the ability of this protease to interact simultaneously and specifically with several consecutive residues upstream of a hydrolyzed peptide bond^37^, thus making it amenable to cleavage. It can be expected that linker rearrangement resulting from interaction of the CTD with the secretory machinery facilitates linker cleavage by PorU sortase. Interestingly, however, fXa was unable to release the CTD from the full-length proRgpB in which the IEGRAA motif was substituted for native residues. By contrast, full-length proRgpB in which the motif was inserted into the linker was susceptible to proteolysis by fXa. This indicates that elongation of the IgSF-CTD interdomain linker results in enhanced susceptibility of the linker region to proteolytic processing.

Finally, the observed propensity of the CTD to form dimers by β-strand swapping may also contribute to recognition of this domain by the T9SS apparatus for OM translocation and A-LPS modification of cargo proteins. The fact that the integrity of the last β-strand of the CTD is essential for secretion of RgpB^14^ lends some credit to this hypothesis, which is also supported by domain/strand swapping events observed previously in Ig-domains closely structurally related to CTD: CD2^44^ and CD4^45^. Furthermore, β-sheet complementation in the Ig-like domains observed in subunit-subunit and chaperone-subunit interactions functions as the mechanism of bacterial pili assembly by the chaperone-usher secretion pathway (T7SS)^46^. Therefore, it is likely that a somewhat similar mechanism may be involved in the secretion and modification of proteins secreted via T9SS in *Bacteroidetes*. This possibility is being currently tested in our laboratories.

## Methods

### Bacterial strains and general growth conditions

*Porphyromonas gingivalis* wild-type W83 and mutants were grown in enriched tryptic soy broth medium (eTSB; per litre: 30 g trypticase soy broth, 5 g yeast extract, 5 mg haemin, pH 7.5 and supplemented with 0.5 g L-cysteine and 2 mg menadione) or on blood eTSB agar (eTSB medium + 15 g agar per litre and supplemented with 4% defibrinated sheep’s blood) at 37 °C in an anaerobic chamber (Don Whitley Scientific, UK) with an atmosphere of 90% N_2_, 5% CO_2_ and 5% H_2_. *E. coli* strain DH5*α* was used for all plasmid construction work and was grown in Luria–Bertani medium and agar. For antibiotic selection in *E. coli*, ampicillin was used at 100 μg ml^−1^. For *P. gingivalis* growth selection on solid media erythromycin was used at 5 μg ml^−1^and tetracycline at 1 μg ml^−1^.

### Recombinant protein expression and purification

The coding sequence of CTD alone (bases 1987–2208) and IgSF-CTD (bases 1729–2208) of *rgpB* (PG0506; NCBI Reference Sequence: WP_010956050.1) were amplified from *P. gingivalis* W83 genomic DNA by PCR using Accuprime Pfx DNA polymerase (Invitrogen, CA, USA) and the following forward primers: RgpBCterBamHIF and RgpBIgBamHIF, and a common reverse primer: RgpBCterXhoIR (Table S3). Amplicons were purified and cloned initially into the pGEX-5T expression vector^47^ using the *Bam*HI/*Xho*I sites to give rise to expression plasmids pRCter-B and pRIg-A, respectively. These vectors have a thrombin cleavage site for removal of the GST tag but we have found thrombin to be promiscuous and cleaved the rIgSF-CTD product at multiple sites. Thus, SLIM site-direct mutagenesis was used to replace the thrombin site of pRIg-A to Prescission cleavage site (using primers RThr2PreFA, RThr2PreFB, GThr2PreRA, GThr2PreRB) to give pRIgP-B. Subsequently, the SLIM method was also used to mutate the wild-type construct of IgSF-CTD in pRIgP-B to produce mutant recombinant proteins with 6×His insertion (r662i6H, r664i6H, r665iH) and substitution of linker residues (r665s6H) or insertion of a factor Xa cleavage site (Ile-Glu-Gly-Arg-Ala-Ala) (r662iXa, r664Xa) and substitution of linker residues (r662sXa, r665sXa). The constructs were confirmed by DNA sequencing and they were transformed into *E. coli* strain BL21 (DE3) (New England Biolabs, Ipswich, MA, USA) under the control of the *lac* promoter. The resulting recombinant products included an N-terminal GST tag, a PreScission protease cleavage site, followed by the cloned protein.

Transformed *E. coli* was grown in Luria-Bertani (LB) media at 37 °C to approximately OD_600_ 0.75 and expression of recombinant proteins were induced by the addition of 0.1 mM isopropyl-1-thio-β-D-galactopyranoside (IPTG). After 16 h of cultivation at room temperature, cells were harvested by centrifugation (6000 × g, 15 min, 4 °C) and resuspended in PBS supplemented with 5 mM EDTA and 0.02% NaN_3_ (15 ml per pellet from 1 L of culture) and subsequently lysed by sonication (cycle of 30 × 0.5 s pulses at amplitude of 70% per pellet from 1 L of culture) using Branson Sonifier Digital 450 (Branson Ultrasonics, Danbury, CT, USA). Cell lysates were clarified by centrifugation (40 000 × g, 40 min, 4 °C), filtered through a 0.45-μm syringe filter, and applied at 4 °C onto a glutathione-Sepharose 4 Fast Flow column (bed volume 5 ml) equilibrated with PBS supplemented with 5 mM EDTA and 0.02% NaN_3_. After washing, 10 ml of equilibration/washing buffer containing 100 μl of PreScission protease stock solution (1 U/ml) was applied and the column was incubated for 40 h at 4 °C before elution. The final purification was accomplished by size exclusion chromatography on calibrated Superdex S75 column using ÄCTA Purifier System (GE Healthcare Life Sciences). Protein concentration in the final preparation was determined by measurement of absorbance at 280 nm using Nanodrop (NanoDrop Products, Wilmington, DE, USA) and BCA assay (Thermo Scientific Pierce). Purity of obtained protein was verified by SDS-PAGE electrophoresis.

### Crystallization and structural analysis

For all aforementioned rIgSF-CTD constructs, crystallization assays were performed using the sitting drop vapor diffusion method, at both 20 °C and 4 °C in steady temperature crystal farms (Bruker). Briefly, reservoir solutions were prepared with a Tecan robot, whereas crystallization nanodrops of 300 nl were dispensed by a Cartesian robot (Genomic Solutions) in 96 × 2-well MRC plates (Innovadyne), at the joint IBMB-IRB High-Throughput Crystallography Platform at Barcelona Science Park. Crystallization hits were improved and scaled up to the microliter range by mixing 1 microliter of protein and reservoir solutions in 24-well Cryschem crystallization dishes (Hampton Research). The rCTD was crystallized using analogous procedures except that crystals were obtained at Małopolska Centre of Biotechnology of the Jagiellonian University.

Suitable crystals for X-ray diffraction were obtained using protein concentrations of 10, 15, 8 and 6 mg/ml for protein variants rCTD, r664i6H, r665sXa and r665i6H, respectively, in 5 mM Tris, 50 mM NaCl, pH 8.0 (for rCTD) and 50 mM Tris, 5 mM EDTA, pH 8.0 (for remaining constructs). The corresponding reservoir solutions were 25% PEG 3350, 0.2 M Li_2_SO_4_, 0.1 M Bis-Tris pH 5.5 (4 °C, for rCTD), 30% PEG 4000, 0.2 M Ammonium Sulfate (with 10% PEG 300 used as additive for r664i6H, and without for r665sXa), and 30% PEG 8000, 0.2 M Li_2_SO_4_, 0.1 M NaOAc pH 4.5 (for r665i6H). For cryoprotection, reservoir solutions further containing 15% (v/v) (r664i6H) and 20% (v/v) glycerol (r665sXa and r665i6H) were used. No extra cryo-protection was used for the rCTD crystals.

Complete diffraction data sets were collected from liquid N_2_ flash cryo-cooled crystals at 100 K (provided by an Oxford Cryosystems 700 series cryostream) at beam lines ID29 (r664i6H) and ID23-2 (r665sXa and r665i6H), respectively on an ADSC Quantum Q315r CCD detector and a Dectris PILATUS 6M pixel detector, of the European Synchrotron Radiation Facility (Grenoble, France) within the “Block Allocation Group Barcelona”. rCTD crystals were measured employing a rotating anode copper source (MicroMax-007 HF, Rigaku) attached to a Rigaku R-AXIS IV++ detector at the Małopolska Centre for Biotechnology of the Jagiellonian University in Kraków.

The diffraction data were processed with programs XDS^48^ (r664i6H), MOSFLM^49^ (r665sXa, r665i6H and rCTD dimer), and SCALA^50^ within the CCP4 suite of programs (Collaborative Computational Project, 1994) (Table S1).

The structure of r664i6H was solved by molecular replacement with program PHASER^51^ using the IgSF domain (residues 348–432) of RgpB from PDB entry 1CVR as searching model. The map obtained was used as input for electron density modification using the ARP/wARP software^52^. The resulting maps were used to manually complete the model using program COOT^53^, which alternated with crystallographic refinement (including TLS refinement) with programs PHENIX^54^, BUSTER^55^ and REFMAC5^56^.

The structures of r665sXa and rCTD dimer were solved by molecular replacement with PHASER using the r664i6H structure as searching model and were completed using iterative manual building and restrained refinement cycles as aforementioned. Through an equivalent process, we could obtain a similar model for r665i6H mutant, but it was finally not further refined because it proved indistinguishable from r664i6H and r665sXa and had poor refinement statistics.

### SAXS data acquisition, analysis and modelling

SAXS data were measured at the Australian Synchrotron on the SAXS/WAXS beamline^57^ with the data collection parameters presented in Table S2. Data were reduced to *I*(*q*) versus *q* (*I*(*q*) = 4πsin*θ*/*λ* and 2*θ* is the angle between the incident and scattered X-rays, *λ* their wavelength) using the software scatterBrain [ http://www. synchrotron. org.au/aussyncbeamlines/saxswaxs/software-saxswaxs]. Intensities were placed on an absolute scale using the known scattering from H_2_O. All samples were prepared as described above under Recombinant protein expression and purification. SAXS measurements were performed on two consecutive elution fractions from size exclusion chromatography (representing the front half and back half of the peak profile, termed fractions A and B) after dilution to a starting concentration of 1.5 mg/mL according to BCA assays. SAXS data were collected on a concentration series representing 100%, 80%, 60%, 40%, 20% and 10% of the starting concentration of each of the four samples (wild type rIgSF-CTD and the r664i6H fractions A and B). All dilutions were made in gel filtration buffer.

A solvent scattering blank was measured on the gel filtration buffer alone. The scattering profiles for wild type rIgSF-CTD and the r664i6H variant were obtained by subtraction of the corresponding solvent blank scattering from the samples (protein + solvent) scattering. There was no discernable concentration dependence to the scattering profiles, nor any difference in the highest concentration measurement from fractions A and B from either wild type rIgSF-CTD or the r664i6H variant. These highest concentration measurements were therefore averaged to improve signal to noise and used for all further analysis. Molecular weight (MW) estimates for the proteins were made using the method of Orthaber *et. al.*^58^. Values for contrast and partial specific volumes were determined using the program MULCh^59^. SAXS data analysis and modeling used the ATSAS program package^60^, with the specific programs used are specified in the Table S2 along with the data ranges used to determine the given structural parameters.

Both constructs were shown to be monodisperse in solution by virtue of the linear log *I*(*q*) versus *q*^2^ plot (Guinier plot, Fig. S5), good agreement between *R*_*g*_ values determined by Guinier or *P*(*r*) analysis (Table S2) and molecular weight estimates that are within 10% of the expected values from the forward scattering intensity (*I*(0)) and from shape reconstructions (modelled using DAMMIF) (Table S2).

DAMMIF modelling calculations were submitted to ATSAS on-line, with P1 symmetry for 20 independent runs that yielded a set of similar shapes for each construct with NSD values all significantly less than 1 as follows (standard deviations in parentheses): rIgSF-CTD (wild type) 0.689(0.032), r664i6H 0.551(0.045). BUNCH modelling used default parameters with the structures for the IgSF (residues 579–662) and CTD (residues 672–736) domains from the crystal structure (PDB code 5AG8), with no structure assigned to the respective linkers for the wild type (residues 663–671 inclusive) and for the r664i6H variant (residues 663–671 inclusive plus the hexahistidine insert) so that the domains were free to adopt positions that optimised the fit to the scattering data. Ensemble Optimisation Modelling (EOM) used the same domain structures and flexible linkers and was run using the ATSAS on-line server with default parameters.

### Plasmid construction for porN-deletion mutant

Two 1-kb flanking regions 5′ and 3′ to the *porN* gene (The Institute for Genomic Research [TIGR] accession no. PG0291 in *P. gingivalis* strain W83; PGN1673 in *P. gingivalis* strain ATCC33277), encoding an essential component of T9SS^7^, were amplified by PCR with PG266FrANdeIF + PG266FrASmaIR and PG266FrBXbaIF + PG266FrBPstIR primer pairs using Accuprime Pfx DNA polymerase (Invitrogen, CA) for cloning into pUC19 plasmid (New England Biolabs Inc., MA). An erythromycin resistance cassette *ermF-ermAM* was amplified (using ermFAMXmaIF and ermFAMXbaIR primers) from plasmid pVA2198^61^ and ligated into the modified pUC19 vector above to give the final suicide plasmid, p266AeB-C, for homologous recombination into the W83 genome to give rise to the ΔPorN strain as described below. Correct sequence and orientation of the DNA inserts in the plasmid were confirmed by sequencing.

### Plasmid construction for rgpB mutagenesis studies

Creation of suicide plasmids carrying the desired *rgpB* mutations for homologous recombination into *P. gingivalis* was performed as previously described^14^. Initially, the erythromycin-resistance cassette (*ermF-ermAM*) in the plasmid pURgpB–E constructed for use in RgpB mutagenic studies^14^ was replaced by a tetracycline-resistance gene *tetQ*, amplified from plasmid pNFD13-2^62^ using the primer pair tetQXmaIF and tetQSalIR. Using a modified SLIM method of mutagenesis^63^, a coding sequence for 6×His tag was inserted at the 3′ end of the *rgpB* gene, to create the master plasmid p6HRgpBt-A. The same methodology was applied to modify the master plasmid with insertion of a coding sequence for factor Xa cleavage site (IEGRAA) at base position 1987 in *rgpB* gene to create the RgpB662iXa6H mutant or replacement of bases 1993–2010 in *rgpB* with the coding sequence for IEGRAA to create the RgpB665sXa6H mutant. All primers are listed in Table S3. DNA sequencing of the pertinent region of mutated plasmids confirmed the correct mutation.

### Generation of isogenic mutants via homologous recombination

Electroporation of the suicide plasmid constructs into wild-type *P. gingivalis* W83 allowed for chromosomal integration of the mutated regions into the *P. gingivalis* genome via a double homologous recombination event as described previously^14^. Resistant clones were selected using antibiotic selective media. Southern blotting with digoxigenin-labeled *ermAM-ermF* or *tetQ* gene probes was used to confirm the presence of only one crossover site in the genome in each mutant (ΔPorN, RgpB665sXa6H, and RgpB662iXa6H, Table 1). Deletion and site-directed mutagenic mutants were further verified by DNA sequencing of the pertinent regions of the genome. The ΔPorN strain was then used to generate double mutant strains (ΔPorNRgpB665sXa6H and ΔPorNRgpB662iXa6H, Table 1) expressing the modified *rgpB* gene in the background of non-functional T9SS system, which retains unprocessed progingipains in the periplasm^7^.

### Purification of His-tagged modified full-length proRgpB

Strains ΔPorNRgpB665sXa6H and ΔPorNRgpB662iXa6H were grown to a stationary phase (OD600 = 1.5) and bacterial cells collected by centrifugation (8,000 × g, 30 min) from 8 L culture. Cells were washed and resuspended in Ni^2+^-Sepharose binding buffer (20 mM sodium phosphate buffer, 500 mM NaCl, 20 mM imidazole, pH 7.4, supplemented with 0.02% NaN_3_ and 1.5 mM 4,4-dithiodipyridine (Sigma-Aldrich)) and lysed by sonication. Insoluble cell debris were removed by ultracentrifugation (100,000 × g, 60 min, 4 °C) and clarified supernatant was applied onto a column of pre-equilibrated Ni^2+^-Sepharose 6 Fast Flow matrix (1.5 ml) (GE Healthcare). After extensive washing, bound proteins were eluted with binding buffer supplemented with 500 mM imidazole. Final purification of proRgpB variants was accomplished by size exclusion chromatography on Superdex 75 column equilibrated with 20 mM Tris, 50 mM NaCl, 5 mM CaCl_2_, 0.02% NaN_3,_ pH 7.5 using the ÄCTA Purifier system. Fractions containing proRgpB were pooled together, concentrated by ultrafiltration (30 kDa cut-off membrane) and dialyzed against 20 mM BisTris, 150 mm NaCl, 5 mM CaCl_2_, pH 6.8 with 0.02% NaN_3_. Protein concentration of the final samples was determined by BCA Assay (Sigma, St Louis, MO, USA).

### Protein cross-linking with glutaraldehyde

The proteins: rCTD, r664i6H and CTD released from proRgpB662iXa at concentrations 1, 0.5 and 0.1 mg ml^−1^ were incubated in PBS with different concentrations of glutaraldehyde ranging form 0.1% to 0.01% for 5 min at 37 °C and then reaction was stopped by addition of Tris to reach final concentration of 100 mM. Next, the samples were subjected to SDS-PAGE and Western blot analysis as described below.

### SDS-PAGE and Western blot analysis

Samples were first boiled in non-reducing SDS-PAGE sample buffer containing 2 mM TLCK for 5 min to inactivate all gingipains prior to the addition of 1% β-mercaptoethanol and boiled for a further 5 min for complete denaturation. Samples were centrifuged briefly at 13,000 × g, 1 min to remove particulates and the supernatant separated on SDS-PAGE and gels were stained with Coomassie Brilliant Blue. For Western blot analysis resolved proteins were subsequently electrotransferred onto 0.22-μm-pore-size nitrocellulose membranes and blocked in 2% (w:v) BSA/PBS solution overnight. RgpB was detected using a 1:2000 dilution of anti-RgpB mouse mAb 18E6 in TTBS (20 mM Tris, 500 mM NaCl, pH 7.5 supplemented with 0.1% Tween 20) for 3 h. Membranes were washed four times with TTBS before being probed for 2 h with a 1:2000 dilution of an alkaline phosphatase-conjugated rabbit anti-mouse polyclonal secondary antibody (Dako Cytomation, Denmark). Development was carried out using the AP Conjugate Substrate kit as per manufacturer’s instructions (Bio-Rad Lab., CA, USA).

The rCTD and CTD released from proRgpB662iXa were detected using a 1:13,500 dilution (1 μg ml^−1^) of anti-CTD rabbit polyclonal antibody in 5% (w/v) skim milk/TTBS solution. Membranes were washed four times with TTBS before being probed for 1 h with a 1:40,000 dilution of an HRP-conjugated goat anti-rabbit polyclonal secondary antibody (Sigma-Aldrich). Development was carried out using the ECL Western blotting substrate kit according to manufacturer’s instructions (Pierce, Thermo Scientific).

### Limited proteolysis investigations

Different rIgSF-CTD variants were incubated with tested proteases in 20 mM Tris, 50 mM NaCl, 5 mM CaCl_2_, pH 7.6 (bovine factor Xa (Sigma-Aldrich, St. Louis, MO, USA)), 25 mM Tris, 1 mM EDTA, 0.02% NaN_3_, pH 8.0 (Lys-C endopeptidase and V8 protease (Promega, Fitchburg, WI, USA)) or 0.1 M Tris, 150 mM NaCl, 5 mM EDTA, 0.05% Tween 20, 0.02% NaN_3_, pH 7.5 (porcine pancreatic elastase (Promega)) and human neutrophil elastase (BioCentrum, Krakow, Poland)) for up to 72 h at different weight ratios. Similarly, the full-length proRgpB forms (RgpB665sXa6H and RgpB662iXa6H) were incubated with factor Xa. At specific time points, aliquots were withdrawn for SDS-PAGE analysis and determination of a cleavage site by N-terminal sequence analysis.

### N-terminal sequence analysis

Samples were resolved on 10% SDS-PAGE gels (T/C ratio, 33:1) and electrotransferred in 10 mm CAPS, 10% methanol, pH 11 in a Semi-Dry Transfer Cell onto a PVDF membrane. Protein bands were visualized by Coomassie Brilliant Blue R-250 staining, excised, and analyzed by automated Edman degradation using a Procise 494HT amino acid sequencer (Applied Biosystems, Carlsbad, CA, USA).

### Miscellaneous

Figures were prepared with PYMOL (The PyMOL Molecular Graphics System, Version 1.7.4 Schrödinger, LLC.). Structure similarities were determined with the DALI program^64^. Experimental model validation was performed with MolProbity^65^. The final coordinates of the rIgSF-CTD mutants r664i6H and r665sXa and of the rCTD dimer are available from the Protein Data Bank (PDB codes 5AG8, 5AG9, and 5HFS respectively).

## Additional Information

**How to cite this article**: de Diego, I. *et al*. The outer-membrane export signal of *Porphyromonas gingivalis* type IX secretion system (T9SS) is a conserved C-terminal β-sandwich domain. *Sci. Rep.* **6**, 23123; doi: 10.1038/srep23123 (2016).

## Supplementary Material

Supplementary Information

## Figures and Tables

**Table 1:** *P. gingivalis* strains and mutants used in this study.

Strain	Relevant genotype	Source
W83	Wild-type	Ref. strain
ΔPorN	porN(Erm^r^)	This study
RgpB662iXa6H	rgpBp.G662_T663insIEGRAA; K736insHHHHHH(tetQ^r^)	This study
RgpB665sXa6H	rgpBp.A666E;D667G;V668R;N670A; K736insHHHHHH(tetQ^r^)	This study
ΔPorN/RgpB662iXa6H	porN(Erm^r^) rgpBp.G662_T663insIEGRAA; K736insHHHHHH(tetQ^r^)	This study
ΔPorN/RgpB665sXa6H	porN(Erm^r^) rgpBp.A666E;D667G;V668R;N670A; K736insHHHHHH(tetQ^r^)	This study
