# Supplementary material for: The outer-membrane export signal of Porphyromonas gingivalis type IX secretion system (T9SS) is a conserved C-terminal β-sandwich domain
Source: Sci Rep. 2016 Mar 23;6:23123. doi: 10.1038/srep23123 (PMC4804311; doi:10.1038/srep23123)

Supplementary information for:

The outer-membrane export signal of *Porphyromonas gingivalis* type IX secretion system (T9SS) is a conserved C-terminal β-sandwich domain

Iñaki de Diego Martinez[1,§,#], Miroslaw Ksiazek[2,3,4,§], Danuta Mizgalska[2], Lahari Koneru[4], Przemyslaw Golik[2], Borys Szmigielski[2], Magdalena Nowak[2], Zuzanna Nowakowska[2], Barbara Potempa[4], John A. Houston[4], Jan J. Enghild[5], Ida B. Thøgersen[5], Jinlong Gao[6,7], Ann H. Kwan[8], Jill Trewhella[8], Grzegorz Dubin[2,3], F. Xavier Gomis-Rüth[1], Ky-Anh Nguyen[6,7,*] and Jan Potempa[2,3,4,*]

Table S1. X-ray data collection and refinement statistics

Protein name	rCTD	r664i6H	r665sXa	r665i6H
**PDB entry**	5HFS	5AG8	5AG9	-
		**X-ray data collection**		
Space group	$P6_5$	$P2_1$	$P2_1$	$P2_1$
Wavelength (Å)	1.5419	0.9724	0.8726	0.8726
Unit cell constants *a*, *b*, *c* (Å), â (°)	37.69, 37.69, 184.15, 90.0	51.47, 61.74, 55.30, 117.8	51.6, 61.8, 54.1, 116.8	52.9, 63.2, 56.7, 117.8
Resolution (Å) [a]	32.7-1.97 (2.17-1.97)	48.9-1.90(2.0-1.90)	38.0-2.11(2.22-2.11)	39.3-2.44(2.57-2.44)
Completeness (%)	100.0 (100.0)	98.6 (91.9)	99.9 (100.0)	95.3 (95.5)
No. of measurements / Unique reflections	58,366 / 10,507	86,628 / 23,941	65,902 /17,553	28,402 / 11,842
R_{merge}	0.121 (0.411)	0.049 (0.270)	0.150 (0.447)	0.158 (0.447)
Average intensity (<I> / s(I))	7.0 (2.7)	20.5 (4.5)	6.4 (2.9)	4.9 (2.2)
Redundancy	5.6 (5.3)	3.6 (3.3)	3.8 (4.0)	2.4 (2.4)
		**Refinement**		
Resolution (Å)	∞-1.97	∞-1.90	∞-2.10	
No. reflections	10,402	23,171	16,650	
R_{cryst} / R_{free}	0.20/0.24	0.16/0.20	0.19/0.25	

No. atoms (protein / ligands / solvent)	944/6/33	2208/37/234	2208/25/254
Thermal-displacement parameters ($Å^2$; protein / ligands / solvent)	39.3/93.2/40.0	23.1/49.9/33.1	19.3/53.5/28.6
R.m.s.d. bond lengths (Å)	0.007	0.019	0.016
R.m.s.d. bond angles (°)	1.12	1.98	1.92
Residues in favoured/allowed regions (%) [b]	95.2/100.0	97.2/99.7	97.6/99.7

[a] Values in parentheses refer to highest resolution shell.

[b] According to MOLPROBITY (Davis *et al.*, 2007, Chen *et al.*, 2010).

R_{free} is the same as R_{value} except that calculated for a set of the data (>500) randomly omitted from refinement.

Chen *et al.* MolProbity: all-atom structure validation for macromolecular crystallography. *Acta Crystallogr. sect. D* **66,** 12-21 (2010).

Davis *et al.* MolProbity: all-atom contacts and structure validation for proteins and nucleic acids. *Nucl. Acids Res.* **35,** W375-W383 (2007).

Evans. "Scaling and assessment of data quality." *Acta Crystallogr. sect. D* **62,** 72-82 (2006).

García-Castellanos *et al.* Three-dimensional structure of MecI : Molecular basis for transcriptional regulation of staphylococcal methicillin resistance. *J. Biol. Chem.* **278,** 39897-39905 (2003).

Kabsch. XDS. *Acta Crystallogr. sect. D* **66,** 125-132 (2010).

Sztukowska *et al.* The C-terminal domains of the gingipain K polyprotein are necessary for assembly of the active enzyme and expression of associated activities. *Mol. Microbiol.* **54,** 1393-1408 (2004).

Weiss. Global indicators of X-ray quality. *J. Appl. Cryst.* **34,** 130-135 (2001).

Table S2: SAXS Data Summary

	rIgSF-CTD (wild-type)	r664i6H
Guinier analysis*		
R_g (Å)	24 ± 1	25 ± 1
qR_g-range	0.5 – 1.18	0.5 - 1.15
Fidelity	0.99	0.98
$P(r)$ analysis*		
R_g (Å)	25.2 ± 0.1	26.0 ± 0.2
d_{max} (Å)	98	97
q-range (Å^{-1})	0.02-0.23	0.02-0.23
total estimate	0.68	0.67
MW from $I(0)$ (Da)	16619	15012
Sample details		
Concentration range measured (mg mL^{-1})	0.2 - 1.7	0.14 – 1.4
MW from sequence (Da)	17030	17849
Ratio of *MW* from $I(0)$ to *MW* from sequence	0.98	0.84
Ratio of *MW* from DAMMIF to *MW* from sequence	0.93	0.94
Partial specific volume from sequence (v, cm^3 g^{-1})	0.741	0.739
Contrast from sequence and solvent constituents ($\Delta\rho$, 10^{10} cm^{-2})	2.790	2.814
Model fitting results		
DAMMIF NSD (std dev)	0.684 (0.031)	0.654 (0.042)
DAMMIF χ^2 range	1.123 - 1.146	1.031 – 1.041
DAMMIF MW (Da)	15800	16700
BUNCH χ^2	1.312	1.287
EOM χ^2	1.04	0.97

* Parameters reported for averaged $I(q)$ vs q of highest concentration measurement for two repeated dilution series; 10 – 100% dilutions. 100% conc. estimates 1.66 and 1.35 mg/mL for rIgSF-CTD and r664i6H, respectively.

Data collection parameters	
Instrumentation	Australian Synchrotron SAXS beam-line
Beam geometry	544 μm x 274 μm

q-range measured (Å^{-1})	0.006-0.33
Exposure time (seconds)	1 s x 24 frames
Temperature (^{o}C)	13.5
Software employed for data reduction, analysis and interpretation	
SAXS data reduction	ScatterBrain
Calculation of expected *MW*, *Δρ* and *v* values	MULCh
SAXS data analysis: extrapolation to infinite dilution, Guinier and *P*(*r*)	ATSAS 2.6.0 SAS Data Analysis
ab initio dummy residue modelling	DAMMIF (via ATSAS on-line)
Atomic structure modelling	BUNCH
Ensemble modelling	EOM (via ATSAS on-line)
3D graphic model representations	PYMOL

Table S3. Primers used for molecular biology work.

Primer name	Sequence (5'-3')
Recombinant protein cloning primers	
RgpBIgBamHIF	TAGGATCCACACTTGTCCCGACCAAAATGC
RgpBCterBamHIF	TAGGATCCACATCTATTGCCGACGTAGCC
RgpBCterXhoIR	AACTCGAGAACAGTCTCTTGGCGTAGTGC
Thrombin cleavage to PreScission cleavage mutagenesis primers	
RThr2PreFA	CTGGAAGTTCTGTTCCAGGGTCCGACACTTGTCCCGACCAAAATG
RThr2PreFB	ACACTTGTCCCGACCAAAATG
GThr2PreRA	CGGACCCTGGAACAGAACTTCCAGATCGCTTTTTGGAGGATGGTCGCC
GThr2PreRB	ATCGCTTTTTGGAGGATGGTCGCC
662iXa mutagenesis primers	
662iXaFl	ATCGAAGGCCGTGCAGCTACATCTATTGCCGACGTAGCC
662iXaFs	ACATCTATTGCCGACGTAGCC
662iXaRl	AGCTGCACGGCCTTCGATACCTTCCACTTTCACATCCTTTATCAC
662iXaRs	ACCTTCCACTTTCACATCCTTTATCAC
662i6H mutagenesis primers	
662i6HFl	CATCACCATCACCATCACACATCTATTGCCGACGTAGCC
662iXaFs	ACATCTATTGCCGACGTAGCC
662i6HRl	GTGATGGTGATGGTGATGACCTTCCACTTTCACATCCTTTATCAC
662iXaRs	ACCTTCCACTTTCACATCCTTTATCAC
662sXa mutagenesis primers	
662sXaFl	ATCGAAGGCCGTGCAGCTGTAGCCAATGATAAGCCTTAT
662sXaFs	GTAGCCAATGATAAGCCTTAT
662sXaRl	AGCTGCACGGCCTTCGATTTCCACTTTCACATCCTTTATC
662sXaRs	TTCCACTTTCACATCCTTTATC
664iXa mutagenesis primers	
664iXaFl	ATCGAAGGCCGTGCAGCTATTGCCGACGTAGCCAATGATAAGC
664iXaFs	ATTGCCGACGTAGCCAATGATAAGC
664iXaRl	AGCTGCACGGCCTTCGATAGATGTACCTTCCACTTTCACATC
664iXaRs	AGATGTACCTTCCACTTTCACATCCT
664i6H mutagenesis primers	
664i6HFl	CATCACCATCACCATCACATTGCCGACGTAGCCAATGATAAGC
664iXaFs	ATTGCCGACGTAGCCAATGATAAGC
664i6HRl	GTGATGGTGATGGTGATGAGATGTACCTTCCACTTTCACATC
664iXaRs	AGATGTACCTTCCACTTTCACATCCT
665i6H mutagenesis primers	
665i6HFl	CATCACCATCACCATCACGCCGACGTAGCCAATGATA
665i6HFs	GCCGACGTAGCCAATGATA
665i6HRl	GTGATGGTGATGGTGATGAATAGATGTACCTTCCACTTTCAC
665i6HRs	AATAGATGTACCTTCCACTTTCAC
665sXa mutagenesis primers	
665sXaFl	ATCGAAGGCCGTGCAGCTGATAAGCCTTATACTGTAGCTGTATCAGG
665sXaFs	GATAAGCCTTATACTGTAGCTGTATCAGG
665sXaRl	AGCTGCACGGCCTTCGATAGATGTACCTTCCACTTTCACATCC
665sXaRs	AGATGTACCTTCCACTTTCACATCC
665s6H mutagenesis primers	
665s6HFl	CATCACCATCACCATCACGATAAGCCTTATACTGTAGCTGT
665s6HFs	GATAAGCCTTATACTGTAGCTGT
665s6HRl	GTGATGGTGATGGTGATGAGATGTACCTTCCACTTTCAC
665s6HRs	AGATGTACCTTCCACTTTCAC
RgpB6H mutagenesis primers	

6HRgpBFA	CATCACCATCACCATCACTAATTCACACTGCAATTCTCTAATAAGG
DelRgpBFB	TAATTCACACTGCAATTCTCTAATAAGGGC
6HRgpBRA	GTGATGGTGATGGTGATGCTTCACTATAACCTTTTCTGTATACG
6HRgpBRB	CTTCACTATAACCTTTTCTGTATACGTCTTGC
Cloning primers	
PG266FrBXbaIF	GCGTCTAGAGTATTCGGACTGCATCT
PG266FrBPstIR	ATTACTGCAGCGTCTGAATCGTGCAAA
PG266FrANdeIF	TACCACATATGACTGTAGCAGCCATTACAC
PG266FrASmaR	ATTAACCCGGGCTCCTATGACTGCTTTGA
ermFAMXmaIF	ATTACCCGGGATAGCTTCCGCTATTGC
ermFAMXbaIR	GCTCTAGACGAAGCTGTCAGTAGTATACC
tetQXmaIF	TATACCCGGGACAACGAATTATCTCCTTAACGT
tetQSalIR	ATTTTGTCGACTTTTATTGCCAAGTTCTAATGCT

Figure S1. The structure-based alignment of the CTD of proteins secreted by *P. gingivalis* via T9SS. Conserved hydrophobic residues in β-strands are highlighted in grey. CTDs predicted from MS analysis of culture media are typed in blue with residues after which proteolysis occurred marked in a bold red font (Veith, P. D., *et al.* Protein substrates of a novel secretion system are numerous in the *Bacteroidetes* phylum and have in common a cleavable C-terminal secretion signal, extensive post-translational modification, and cell-surface attachment. *J. Proteome Res*. **12,** 4449-4461 (2013)).

```
                                 s1      s2            s3          s4                  s5          s6            s7
                    ............>>>>>..>>>>>>......>>>>>>.....>>>>>...........>>>>>>...>>>>>>>>.....>>>>>>>..
PG0506    VKVEGTSIADVANDKPYTVAVSGKTITVESPA---AGLTIFDMNGRRVATAK---------NRMVFEA-QNGVYAVRIAT-EGKTYTEKVIVK--
PG0182    ARFRLSYGCDENVDDSHVVSTNGREIIILNQDALDCTVTLFTIEGKLLRRLK---VLAGHREVMKVQT--GGAYIVHLQN-AFTNDVHKVLVEY-
PG0183    FRLSYDEEWVESAEVSVLVGTAGKRIVITNNSEHACQANVYTTDGKLLIRLD----VKPGSKSMTEPL-VDGVYVVSLQSPATSSNVRKVVVN--
PG0193    RNRRQQELQDIQTRYQQSYQTMQEDLQKRQQQ---LFAPIQQKVADAIKKVG-DEENCAYIMEAGMML-YTGATAIDLTA----KVKAKLGIK--
PG0232    ILDDSVEDIVAQTGIVIRPQNGTKQILIEANAAI-KAIVLYDINGRVVLKTT----PNQLRSTVDLSILPEGIYTINIKT-EKSARTEKIHIG--
PG0350    PYFPGITALISIEGESEYSVYAQDGILYLSGMEQGLPVQVYTVGGSMMYSSV------ASGSAMEIQLPRGAAYVVRIG-----SHAIKTAMP--
PG0410    ISISPNPAKAVVTIIYYTDNPSCSVIKIYG-----INGASADITGLPKHLSE-----GYYSIQFNTSNFDPGFYLVTLNVDQKIIDTEKLRIK--
PG0411    YTITSLDNIQSDTSLKIYPNPASYVVRIEGLSRSKSTIELYNALGICILREE----THSEKTEIDVSRLNDGVYLIKVVG-GNKTTTEKVEIKRP
PG0495    KSNAVGEVDTKGFHVYPIPTSKDLTIEIPAEMVG-KVASLIDMNGQIVYRVT----LNNIFQQIDISH-LKGVFLLQIG-----DITERVIVQ--
PG0553    GISNGVAQIENNNAVVAYPSVVTDRFSIKNAHMV-HAAALYSLDGKQVRSWN----NLRNGVTFSVQGLTAGTYMLVMQT-ANGPVSQKIVKQ--
PG0611    TNCYPLSTKPVAGDDEVFVKQQGRQIEIDSNSPI-VQVVVYDLEGKSVFRKR----MTENAYTLSFRAPMLGFMTIMIET-QNSIINKKLNVTQL
PG0614    SCHTTDSQTVVPSSNDINVYIQGTTIGIKAEKLI-KSVYIYDMAGRMLFATS-----QTQGREFCIDLKTKGHILVTVLFA-DNTQTSKNIIL--
PG0616    IINAGQESLDKAEPTATEQIVATPSVKAYVQ----NGKIVVEY-SKMEVFNA-------TGQLVKNESLVPGVYVVRITA-NGVMYFLKVLVP--
PG0626    ILNYQSLQEVEQEGIRIYPNPTTDRLMLDNVGDV-SAIRIIDMYGRPLVVVG---NAGSSKLSIDVSDLGRGNYVVDMQC-KGHRKISRLITKE-
PG0654    MQGNALTDVAVNESIKIYPRPATDFLRIEGSQL--LRLSLFDMNGKLIRATE----LTGDLAIIGVASLPRGTYIAEITAANSKTIRAKVSLR--
PG1030    KESFITSFISPTVVQGVDVYTLAGKIRIESETPV-SEVLLFDLAGRMVLRQTIDNKIYSDIDTNGLKR--SGIYVVSVRLSSGQVFSHKVQV---
PG1326    NIVVANSANIYGADKPFALTVVGKTIVASAFKG--EEITLYDIRGRLIASGC---------DTLRYKA-ENGFYLIKIQV-NGTVYTEKIQIQ--
PG1374    SPTSNLAVDAPTVRIYPNPVGRYALVEIPESLLG-QEAALYDMNGVKVYSFA----VESLRQNIDLTHLPDGTYFFRLD-----NYTTKLIKQ--
PG1424    TNTCTVTGAAKALRAWFNAGRSELAVSVSLNIAGTYRIKLYNTAGEEVAAMTKELVAGTSVFSMDVYSQAPGTYVLVVEG-NGIRETMKILK---
PG1427    GTAVEAIESSEEIRVFPNPARDYVEISAPCIPQE-TSIILFDLSGKIVMKNS----LSAGHGRMDVSRLPNGAYILKVD-----GYTTKINIVH-
PG1548    SSSDIAGKDVSTIVLYPNPAHDYVHVAIPPTYAG-STLRLFDIQGRMQLSTK----IESADMRLDVERLPKGTYIVVVE-----DMVGKLFIR--
PG1795    VHLKKGEGVEAVLTNDKANAYVQNGVIYVAGANG-RQVSLFDMNGKVVYTGV----------SETIAAPQKGMYILRVG-----AKSIKLAI---
PG1798    KALTSMATPSTEAQVAVYLNPSTDRLVILANGI--THLSMYDLQGKLIRDCA----LSGDKVEMGVGSLTKGTYLLKVNT-DQGAFVRKVVIR--
PG1837    ATLNITSLADVTAQKPYTLTVVGKTITVTCQ----GEAMIYDMNGRRLAAGR---------NTVVYTA-QGGHYAVMVVV-DGKSYVEKLAVK--
PG1844    ATLNITSLADVTAQKPYTLTVVGKTITVTCQ----GEAMIYDMNGRRLAAGR---------NTVVYTA-QGGHYAVMVVV-DGKSYVEKLAVK--
PG2024    VDYIPDGVADVTAQKPYTLTVVGKTITVTCQ----GEAMIYDMNGRRLAAGR---------NTVVYTA-QGGYYAVMVVV-DGKSYVEKLAIK--
PG2100    LDVGDVLQKEGSMKLYPNPAKEYVLINLPKEGG--HEAVVYDMQGRIVEKVS--FSGKEYKLNVQYLS--KGTYMLKVVA-DTEYFVEKIIVE--
PG2102    NGVEDIVMQEGSMKLYPNPAQEYAVISLPTAAN--CKAVVYDMQGRVVAEAS----FSGNEYRLNVQHLAKGTYILKVVS-DTERFVEKLIVE--
PG2172    KGGGTGLTNIGLGRIALIQSGNTCTLQYNSNGKR-LALEVYNLLGVKVFTSQ--LPAGSGSYTLPVRL-QRGVHIFRITEGGKPAFVQKYLIK--
PG2198    VTVTNSSLSNVDGQAPYTLRVEGKKIIAEAHG---MEITLYDINGRTVAVAP---------NRLEYMA-QTGFYAVRFDV-GNKHHVSKIQVR--
PG2216    DNKPVITSLAAPISHEIRIWATAGRIFIAGAPAG-TSVQVYDMQGHRIYNAA-----VLADHDIAVAS---GVYVVRAG-----DSTAKVIVPPG
                                   -------      -----------------          -------  ----------    ---------
                                   Motif A           Motif B               Motif C    Motif D        Motif E
```

Figure S2. Superposition of the *ab initio* bead models and BUNCH atomistic models (cartoon representation of domains only) for the **A.** wild-type rIgSF-CTD and **B.** r664i6H variant**.** These models represent the best-fit average models to the data and show the two domains well separated in space with no significant interaction surface.

A.

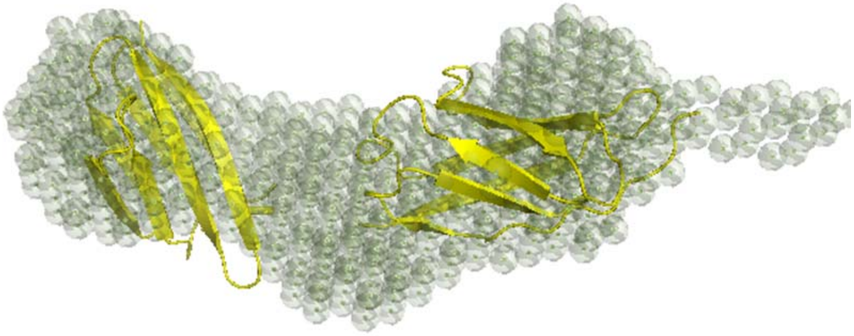

B.

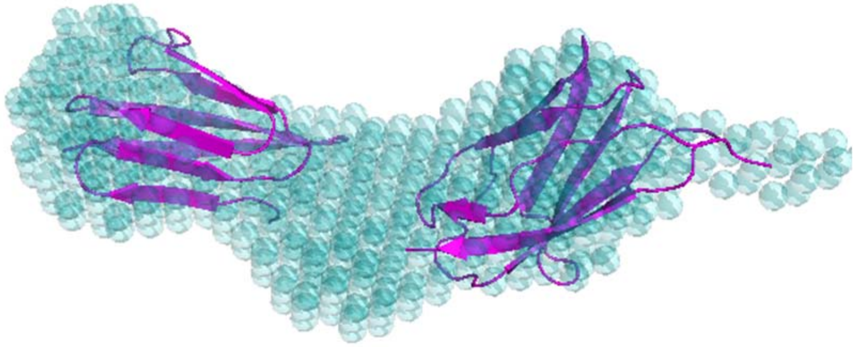

Figure S3. R_g (upper panel) and d_{max} (lower panel) distributions for the ensemble optimisation modelling (EOM) of wild-type rIgSF-CTD (black line) and r664i6H (red line) variant.

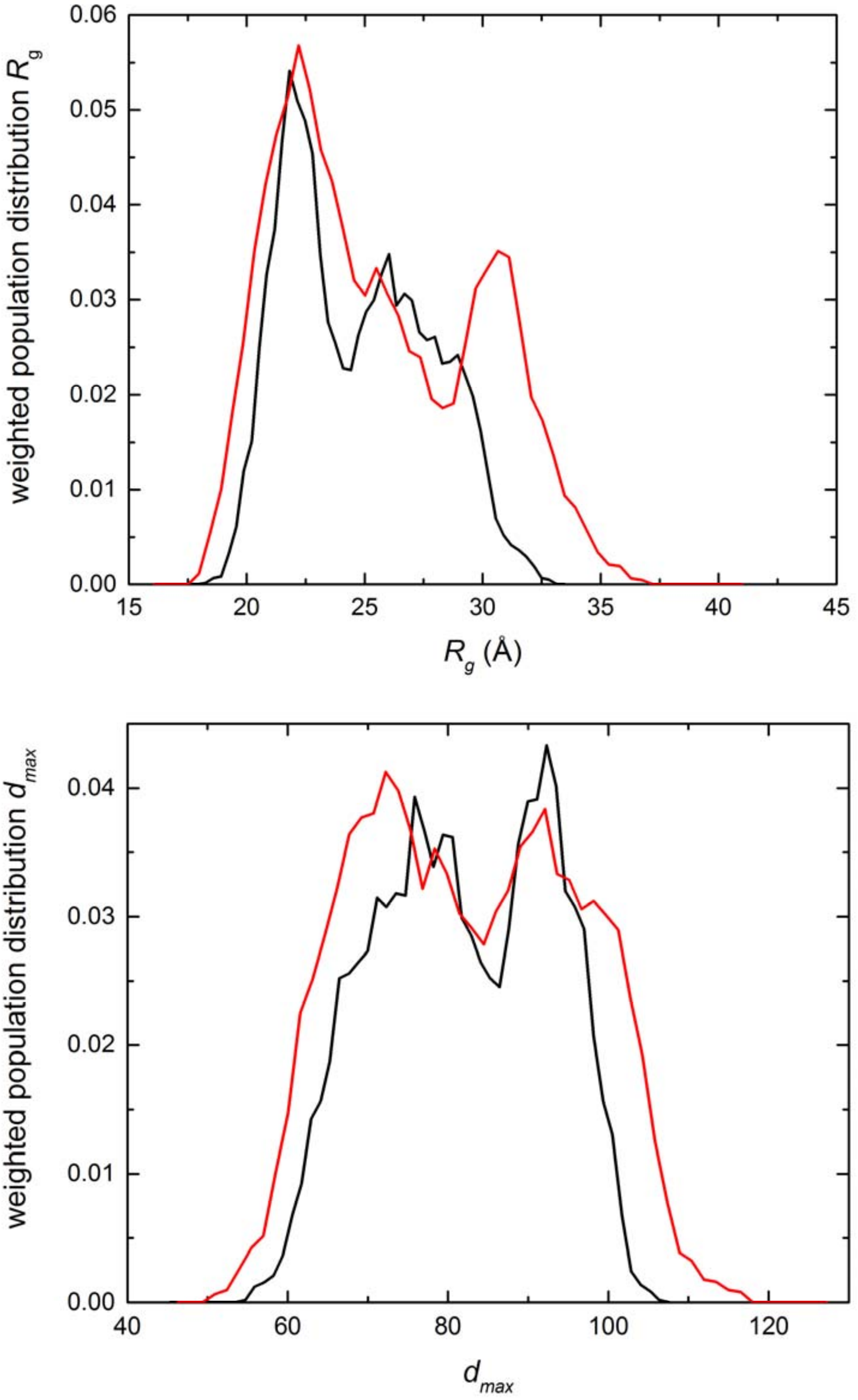

Figure S4. Comparison of RgpB phenotype expressed by wild-type *P. gingivalis* W83 and mutant strains with insertion (RgpB662iXa6H) or substitution (RgpB665sXa6H) of hexapeptide (IEGRAA) in the linker between IgSF and CTD domains. The whole culture (c) and spent growth medium (s) were subjected to Western blotting analysis using RgpB specific mAbs.

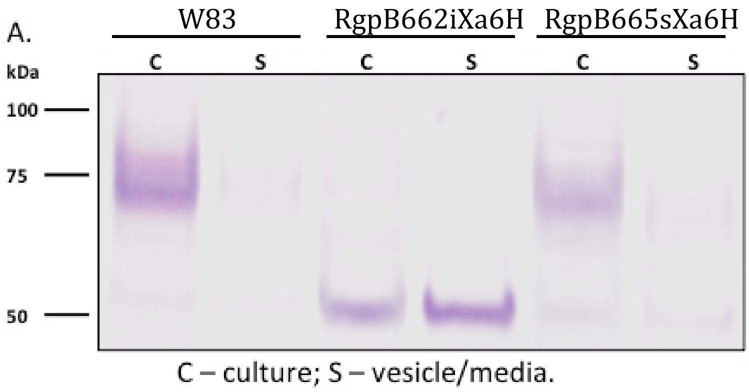

Figure S5. Guinier plots for the SAXS data for wild-type rIgSF-CTD ($qR_g < 1.18$, black symbols) and r664i6H ($qR_g < 1.15$, red symbols) variant. The solid lines are linear fits to the experimental data which shows the expected good fit for solutions of mono-disperse proteins.

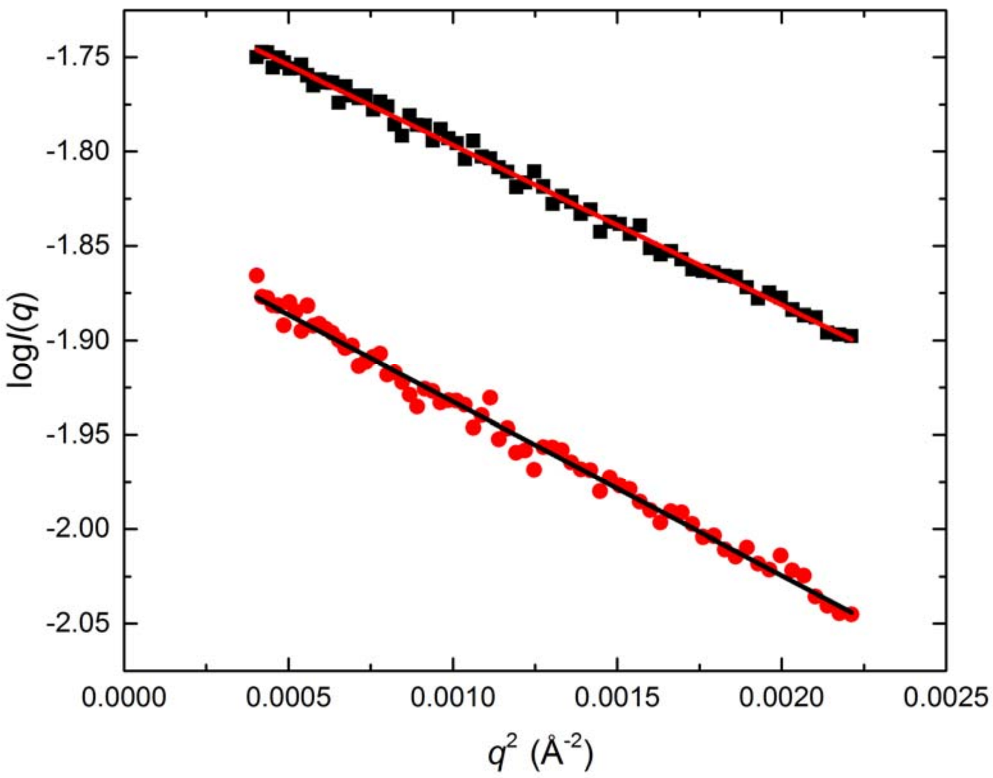

Supplement: Supplementary Information [file srep23123-s1.pdf]
